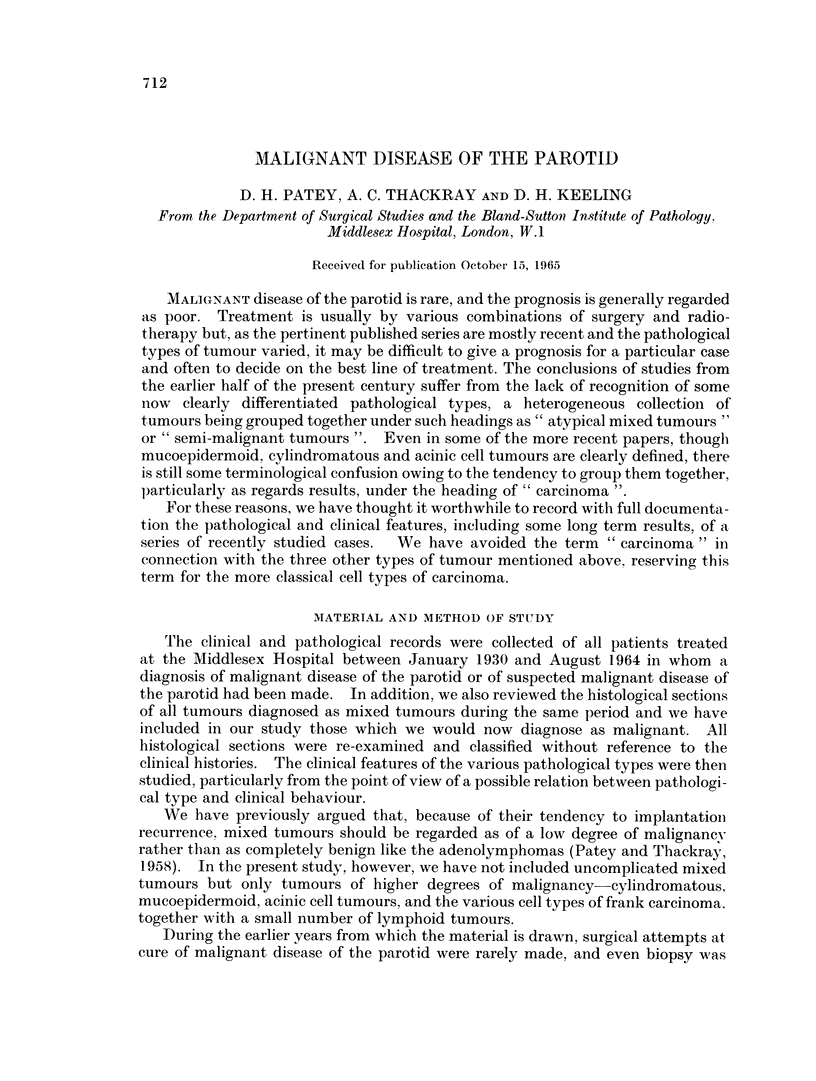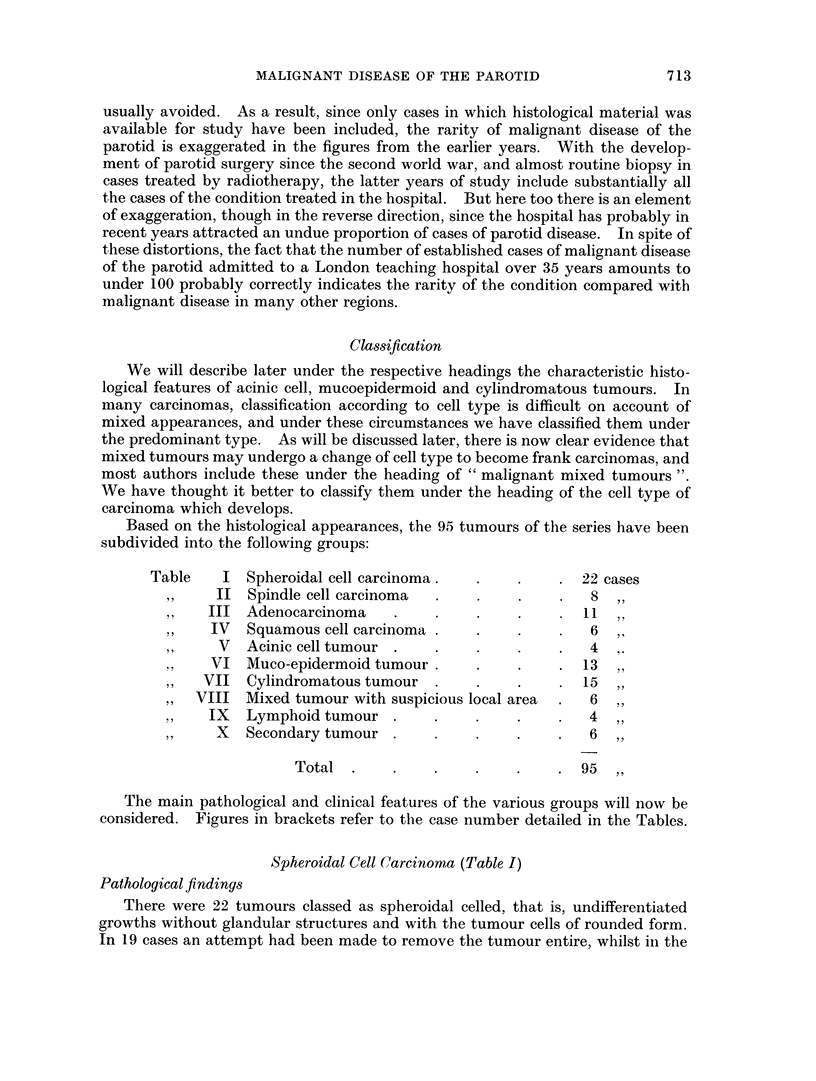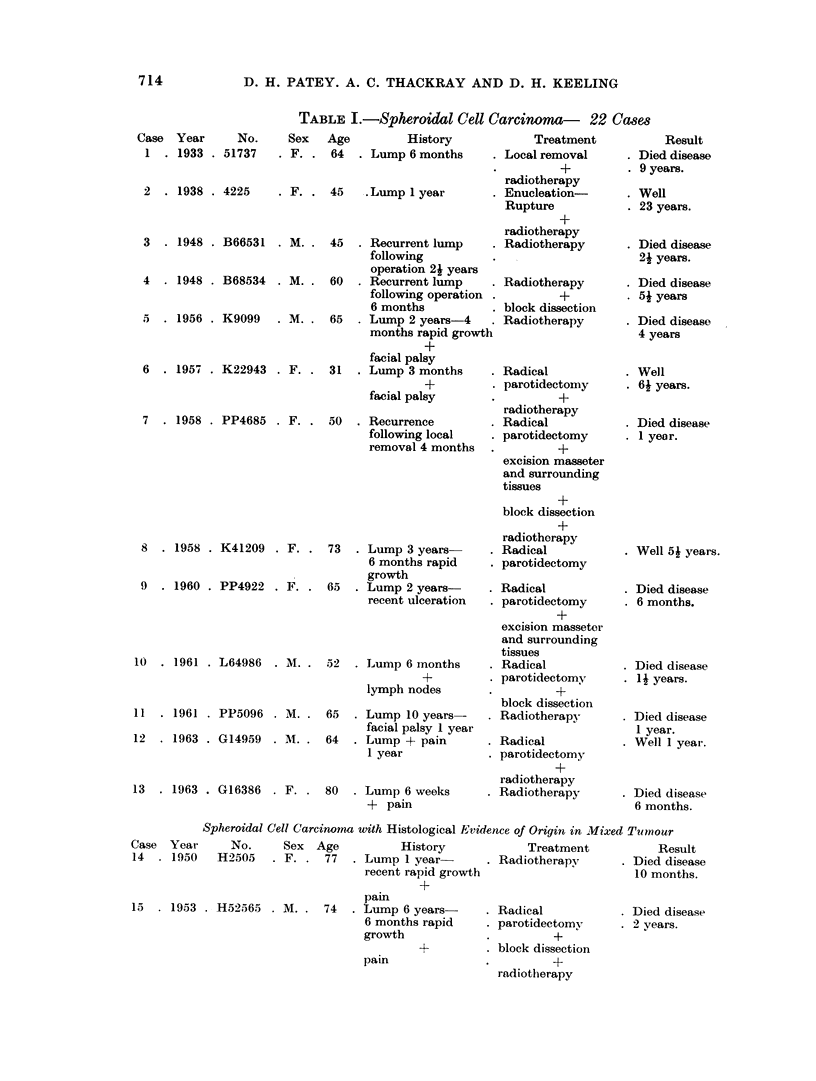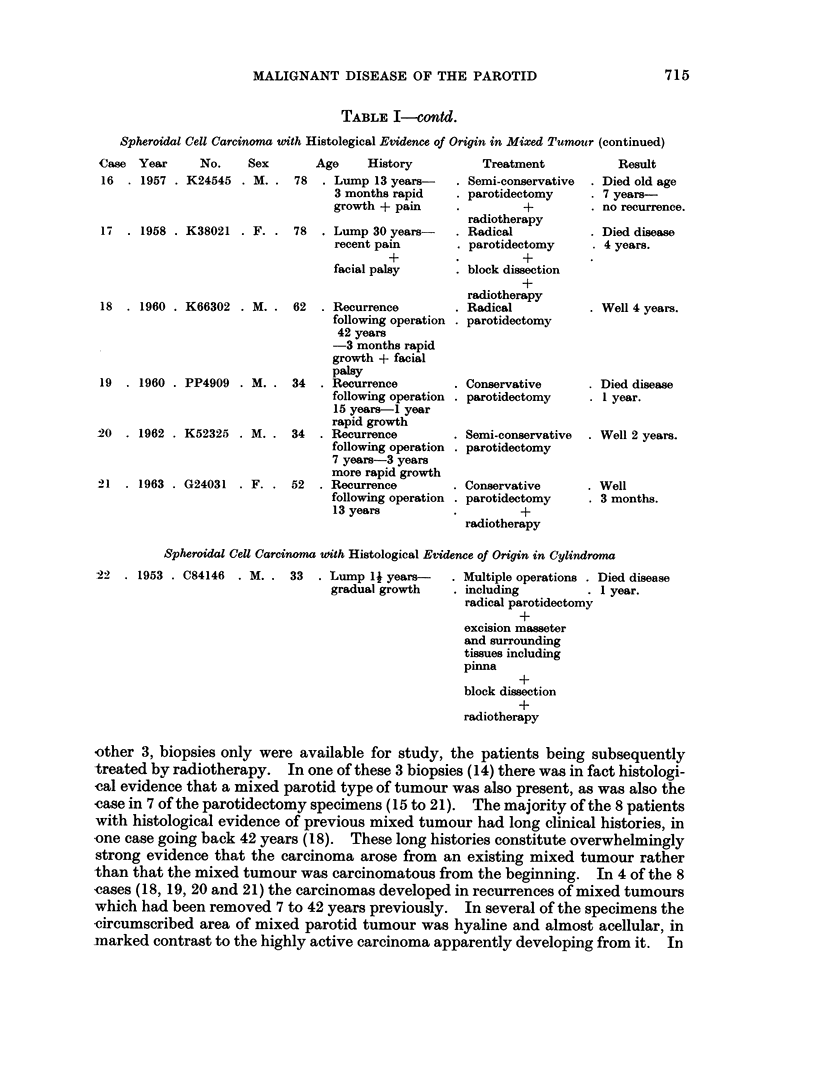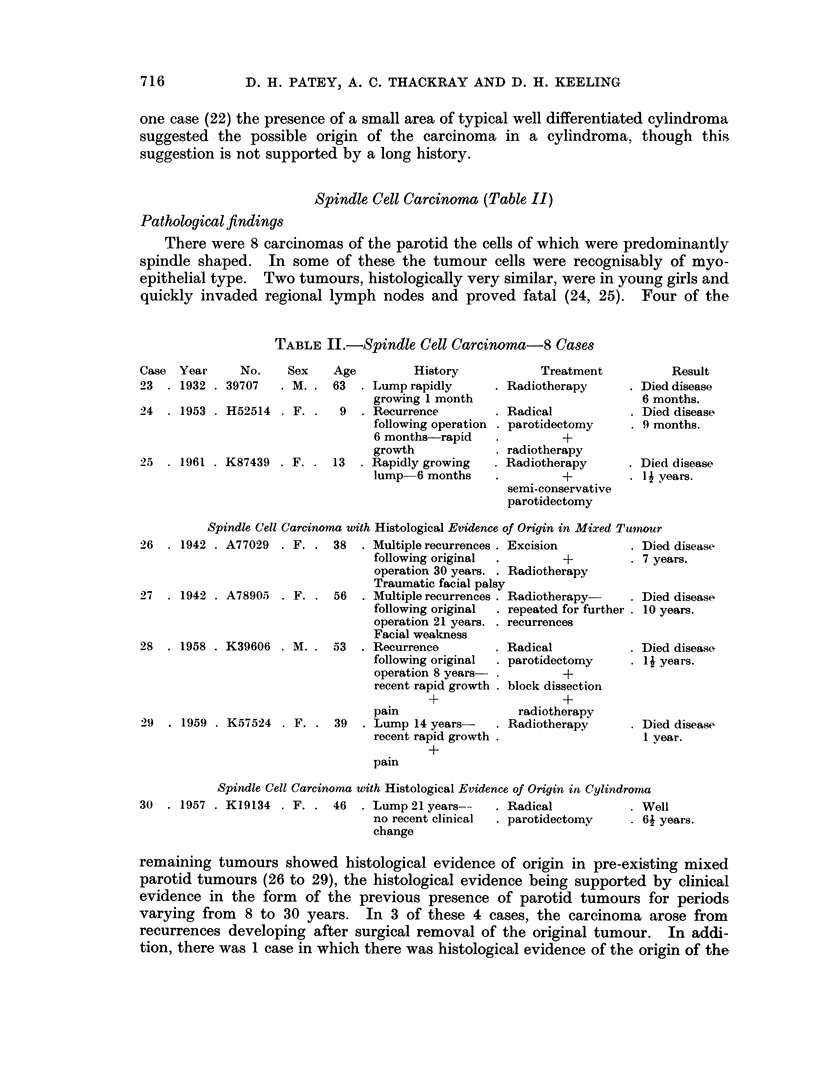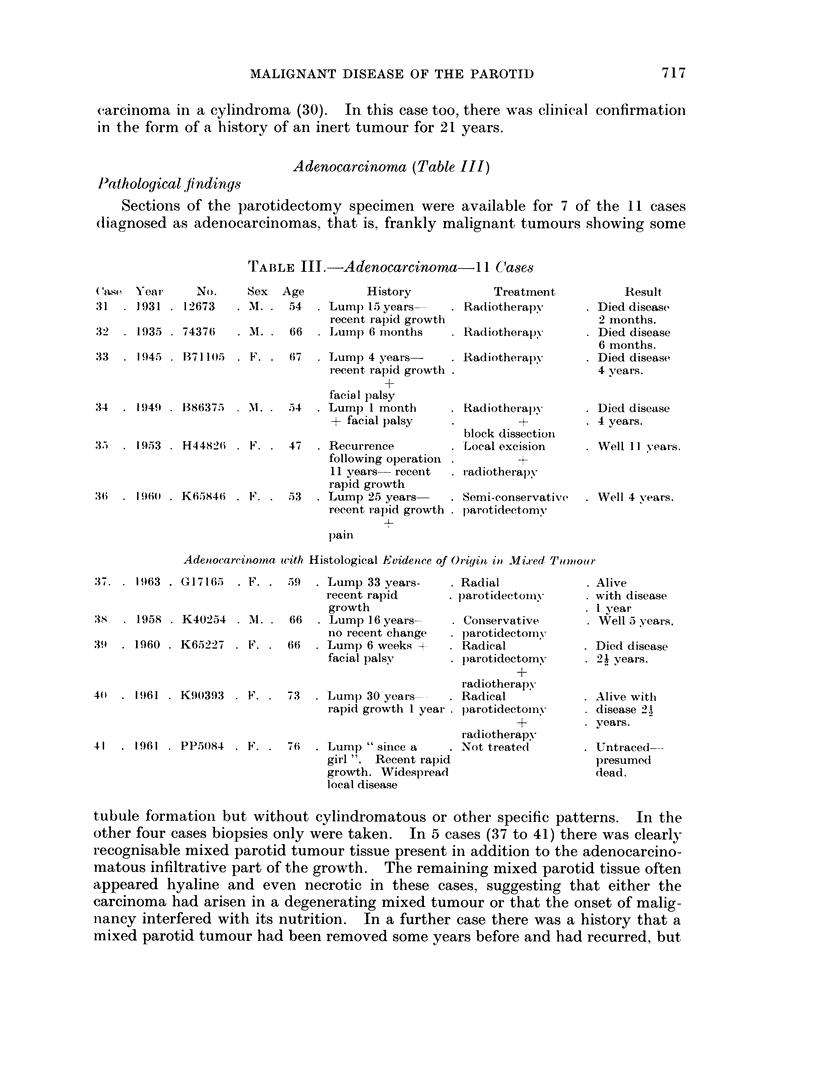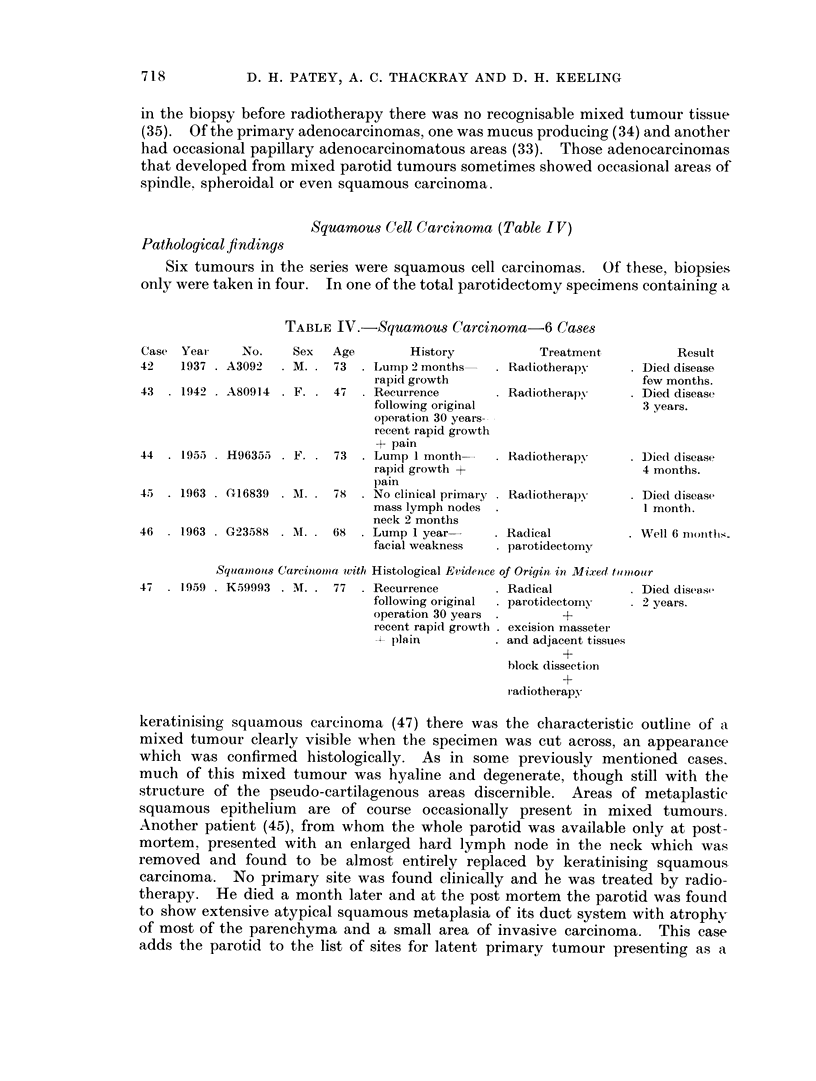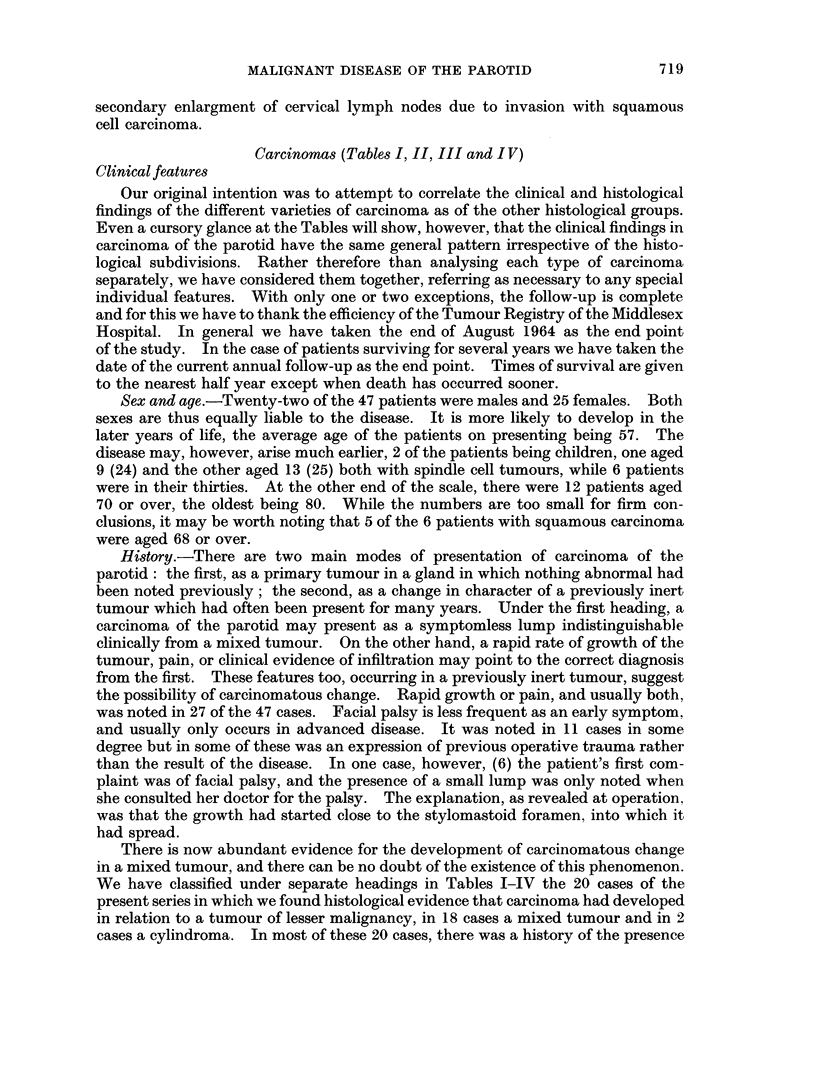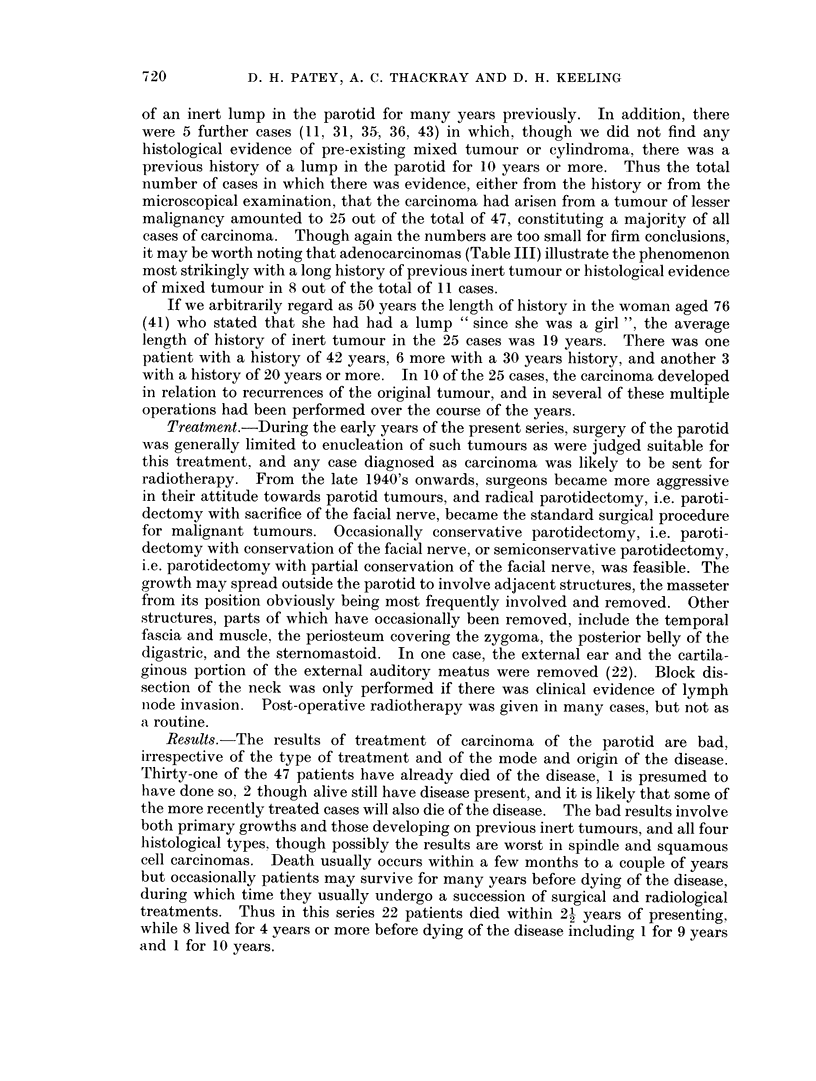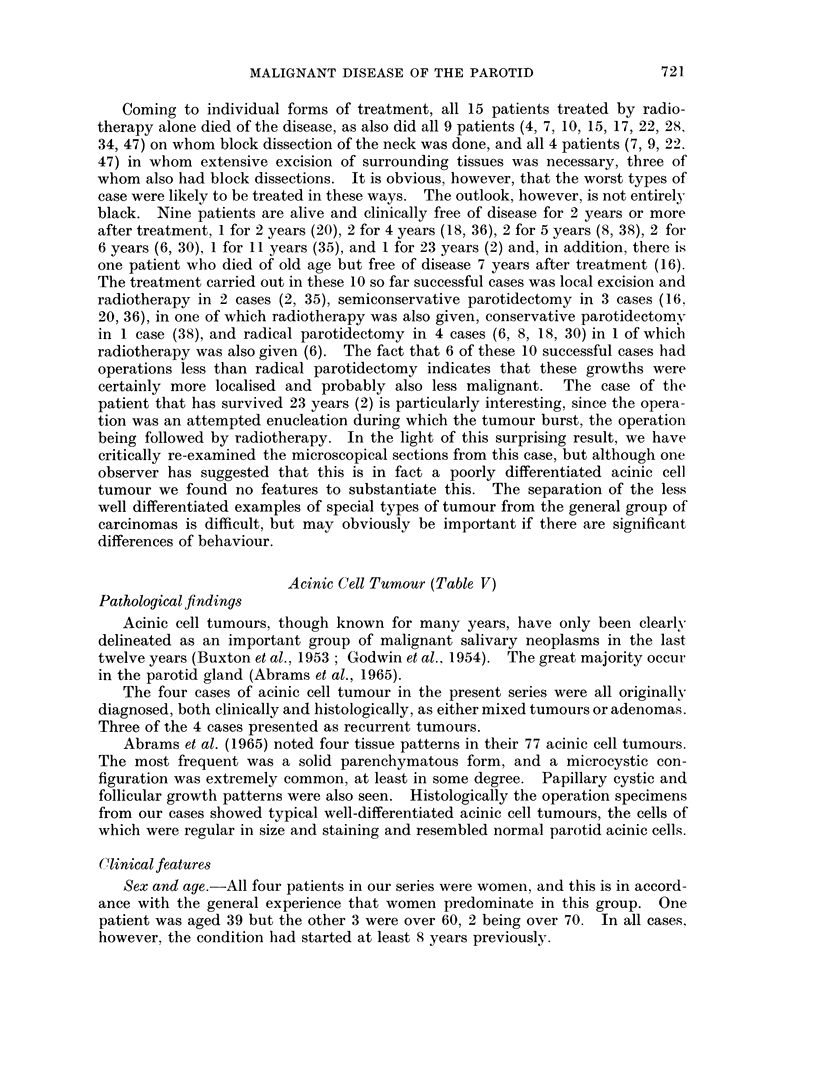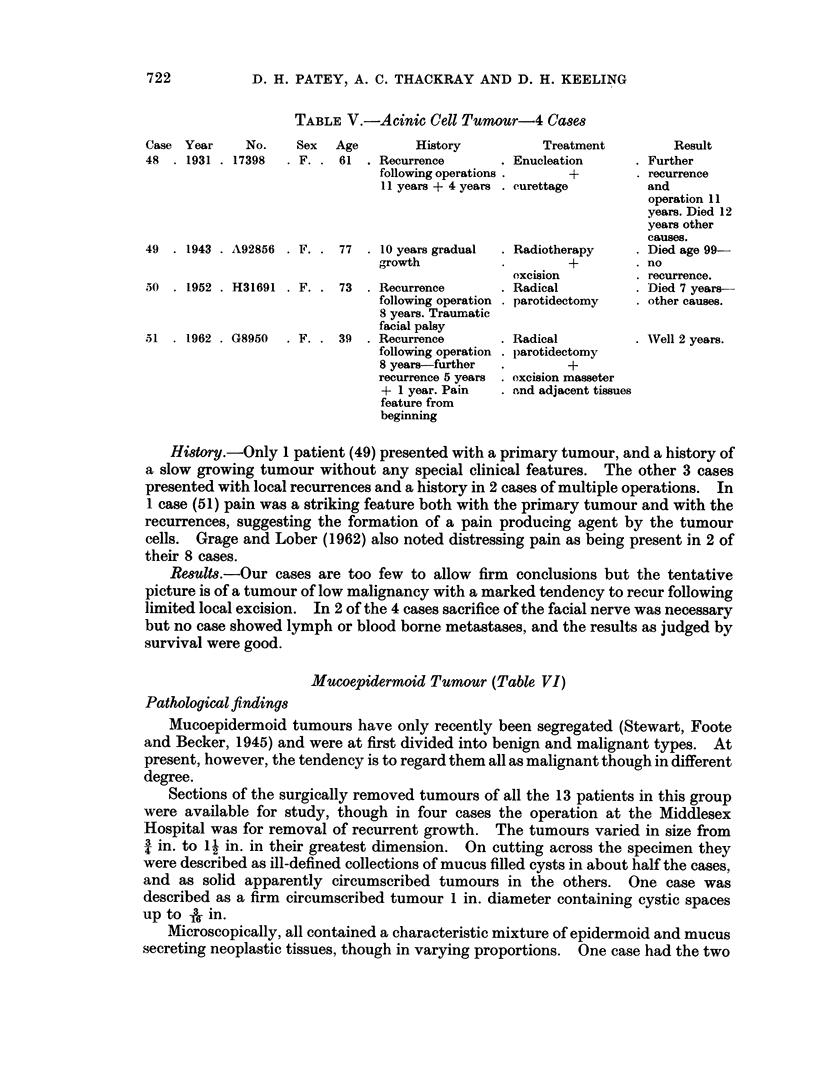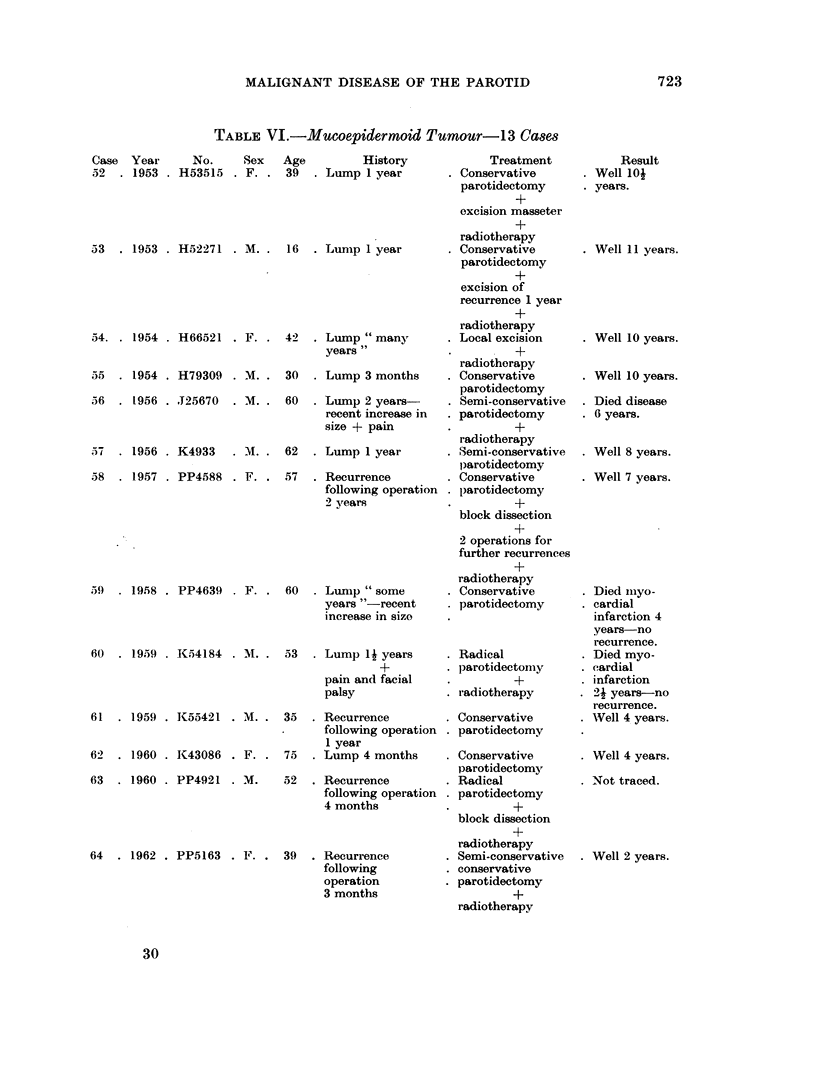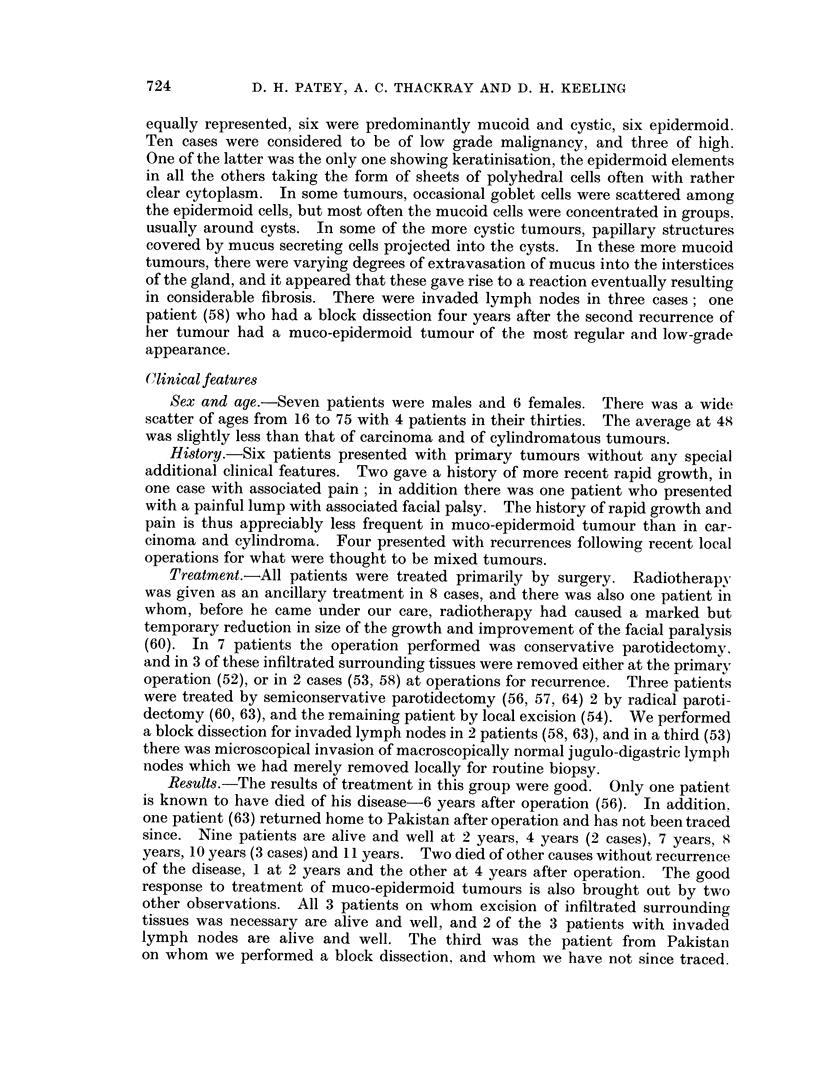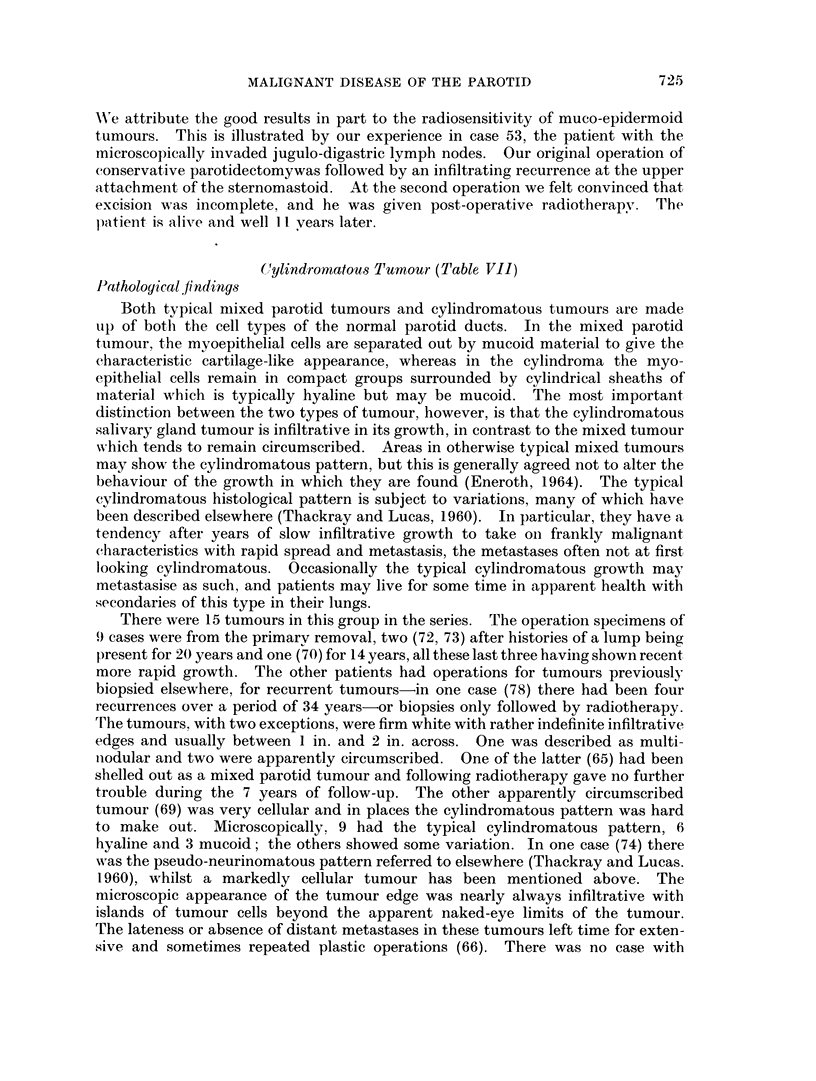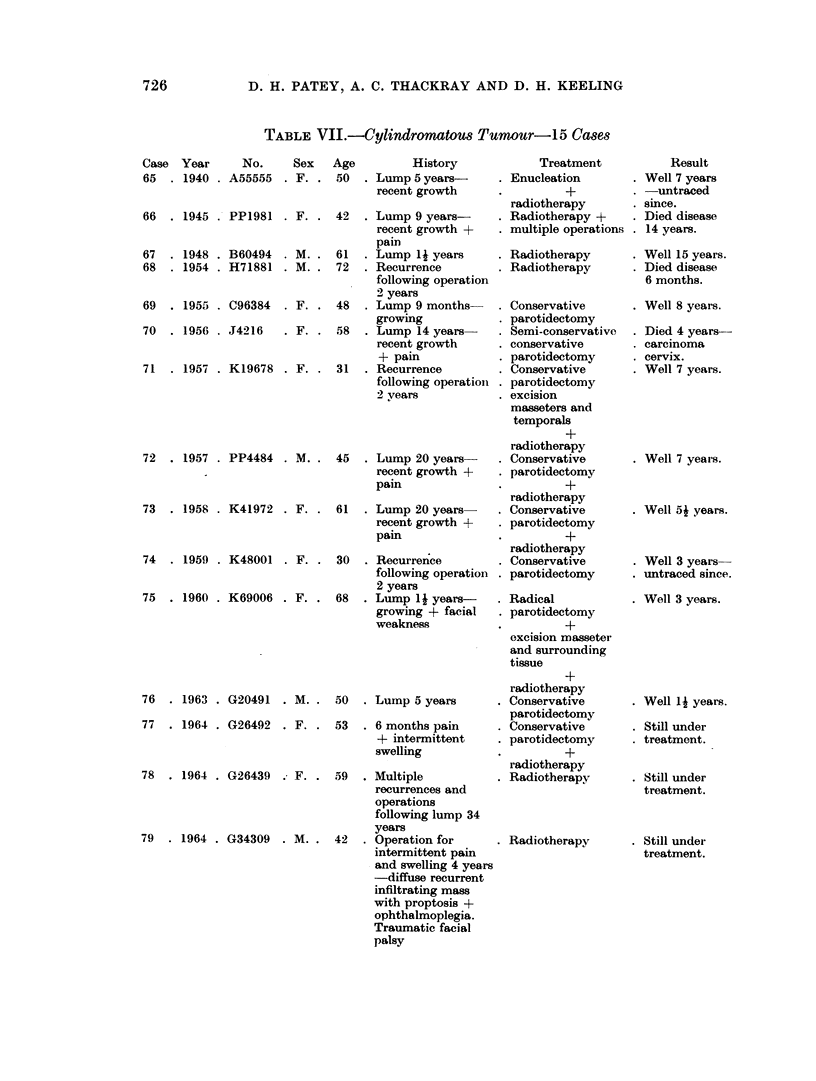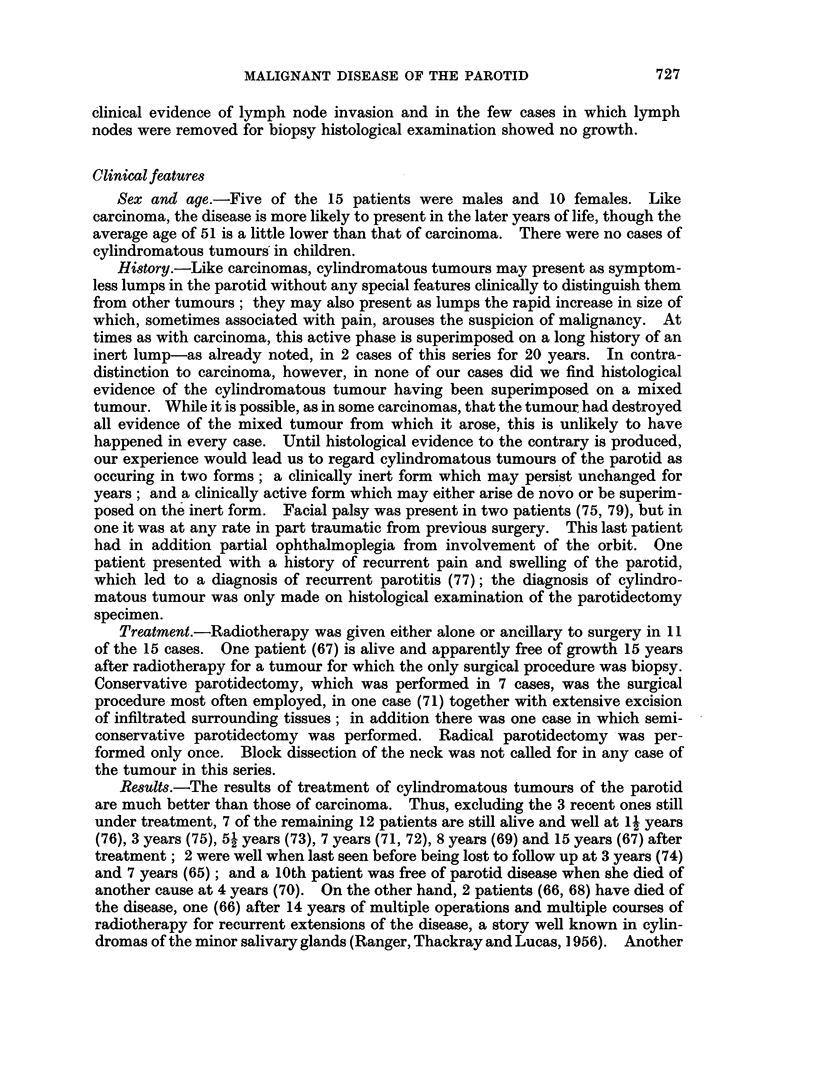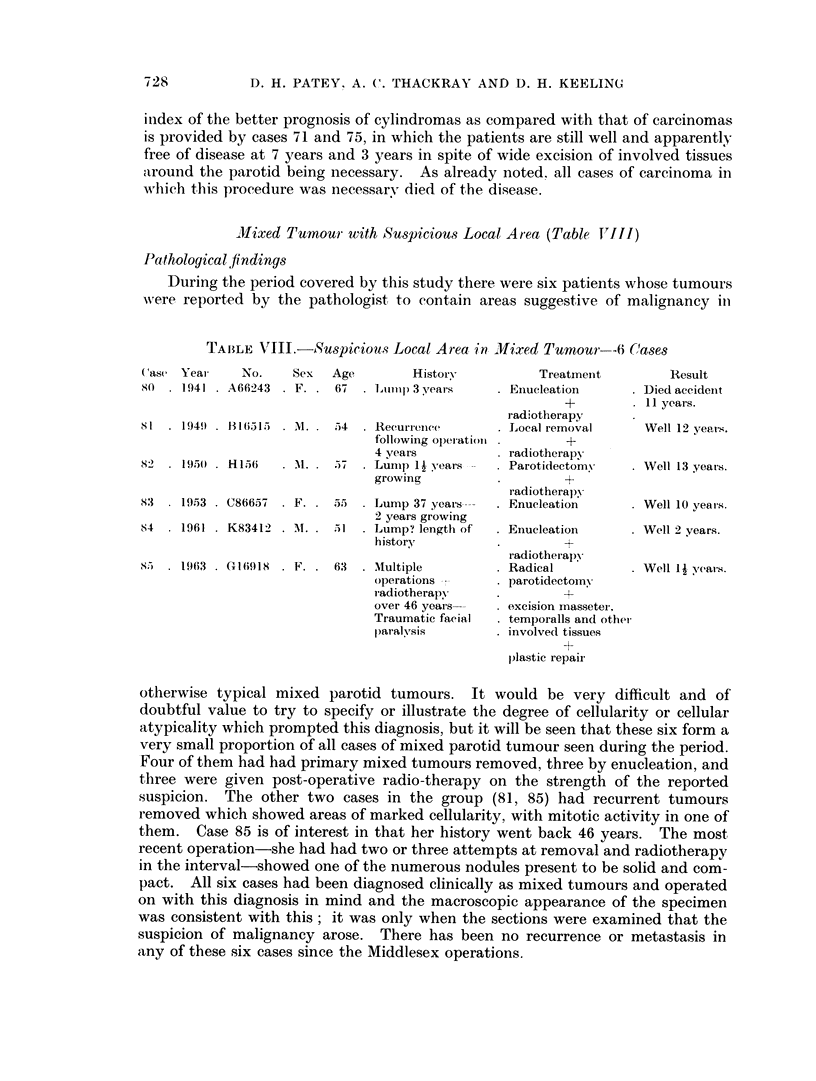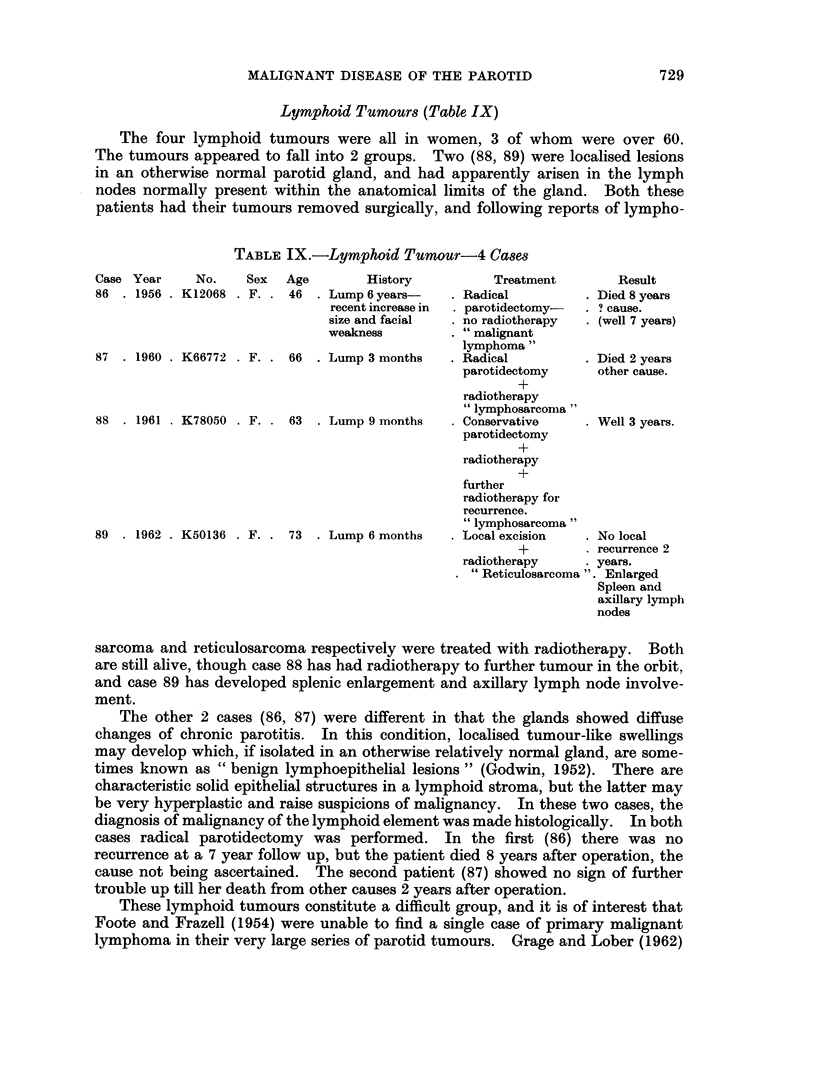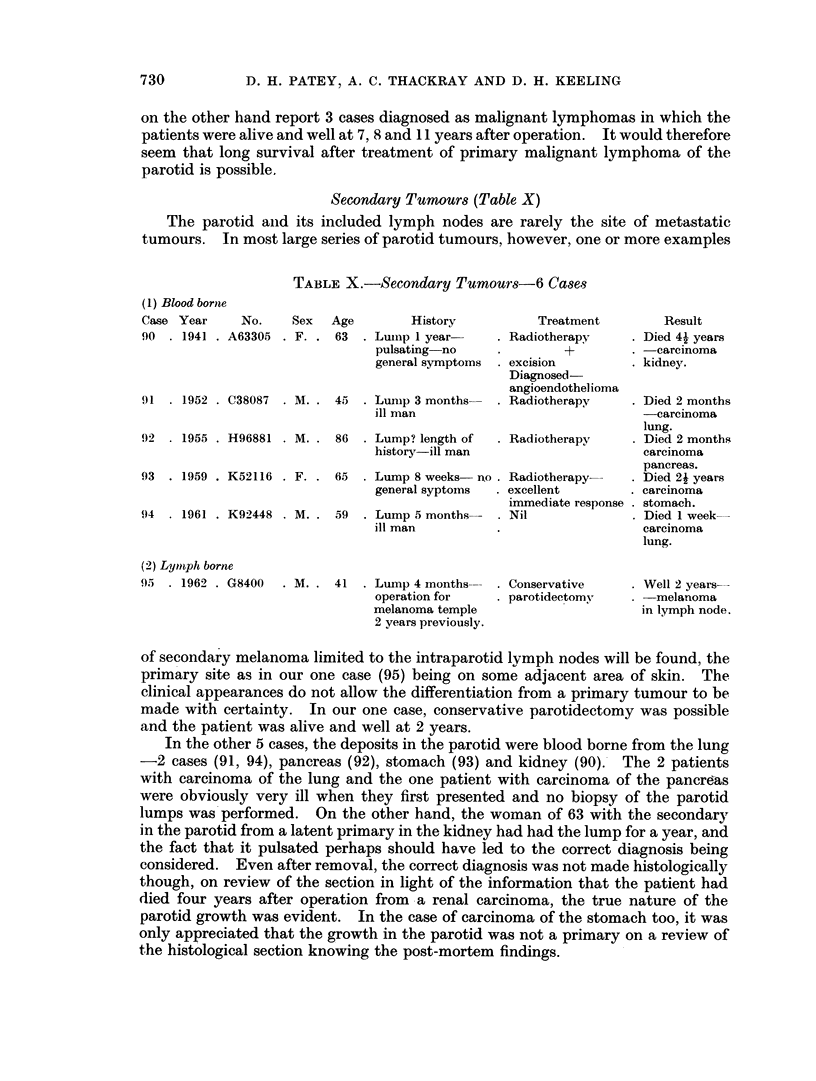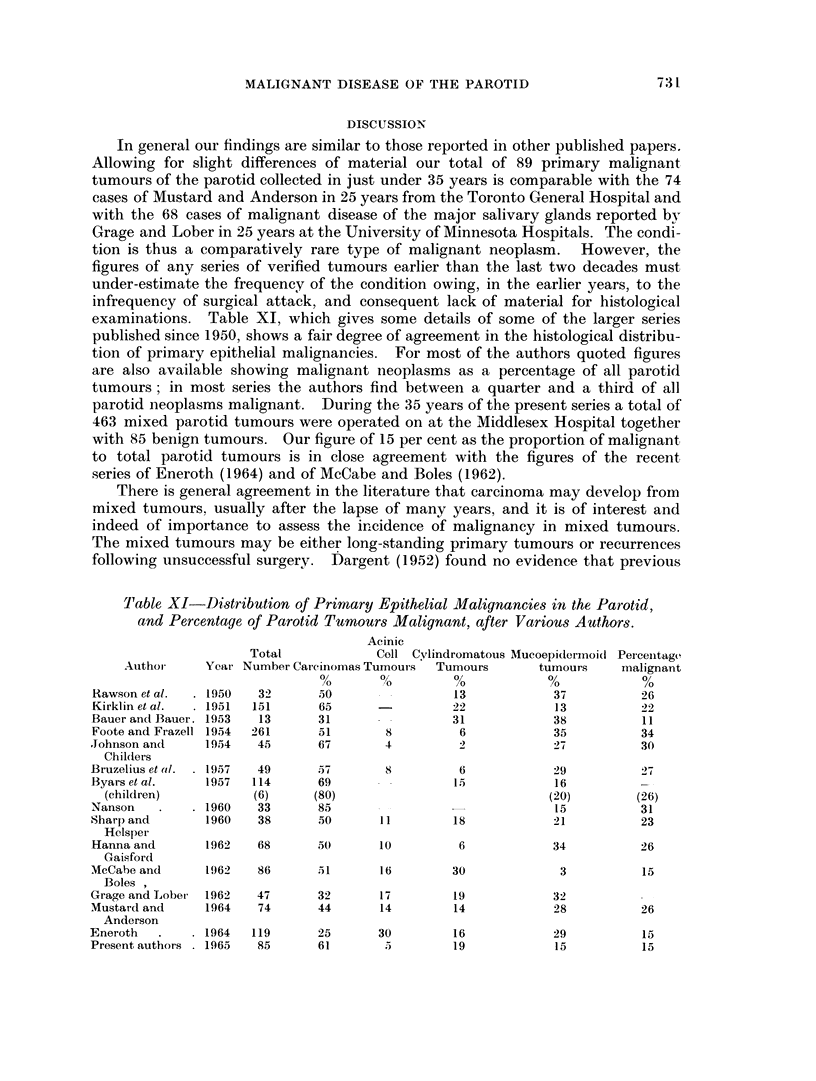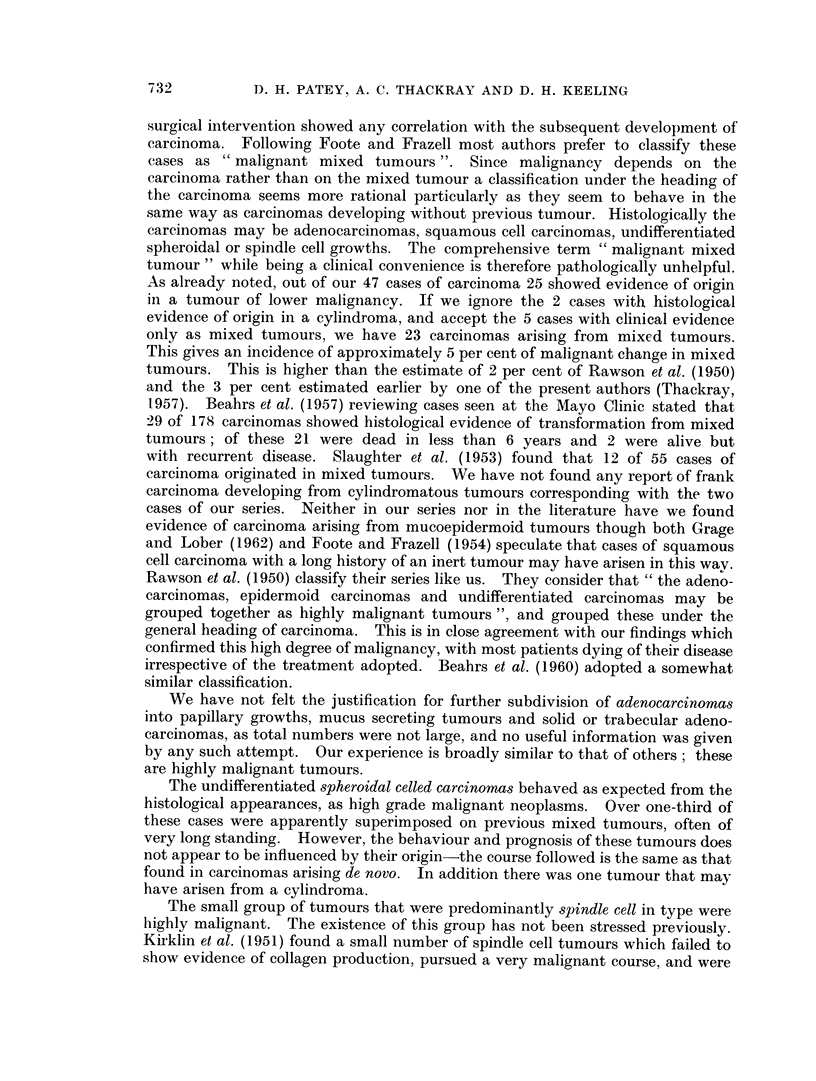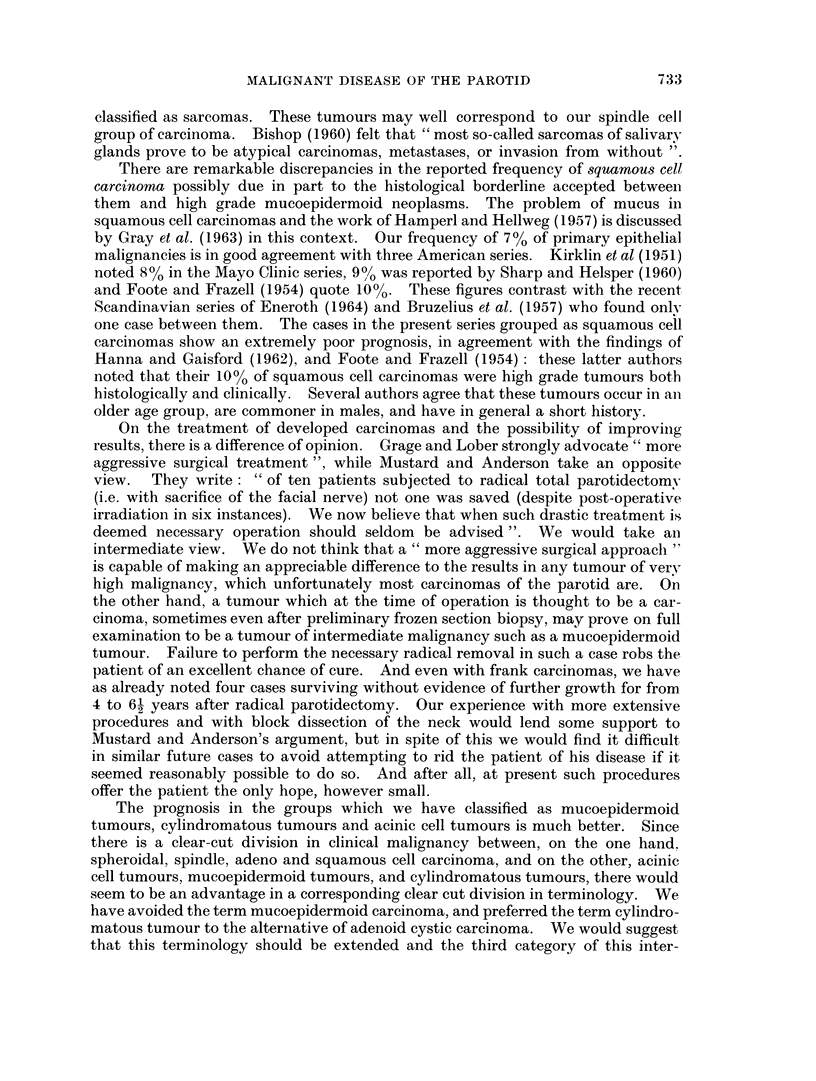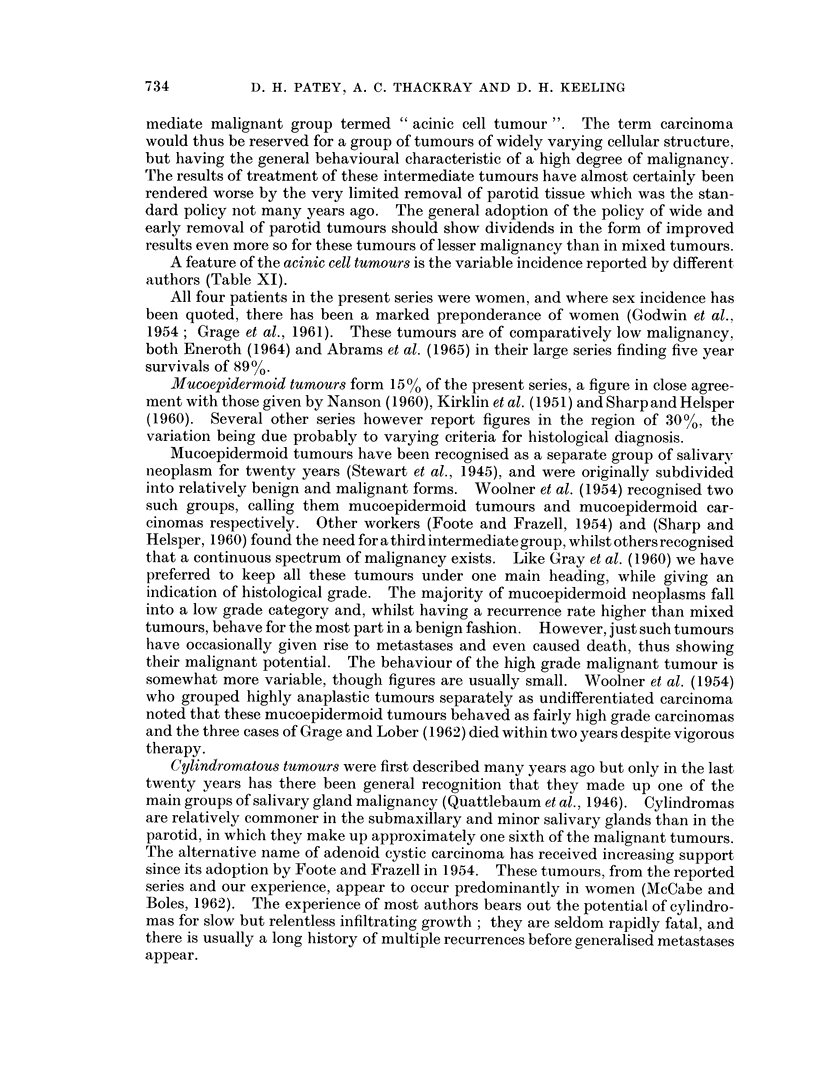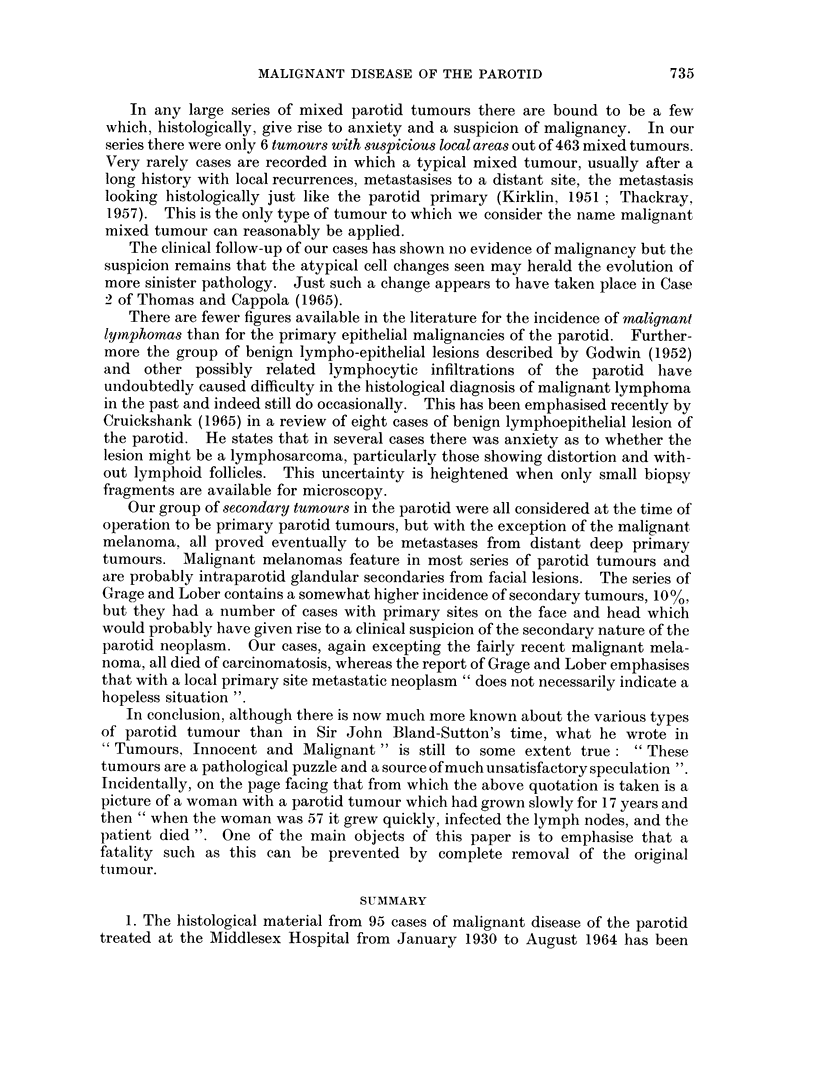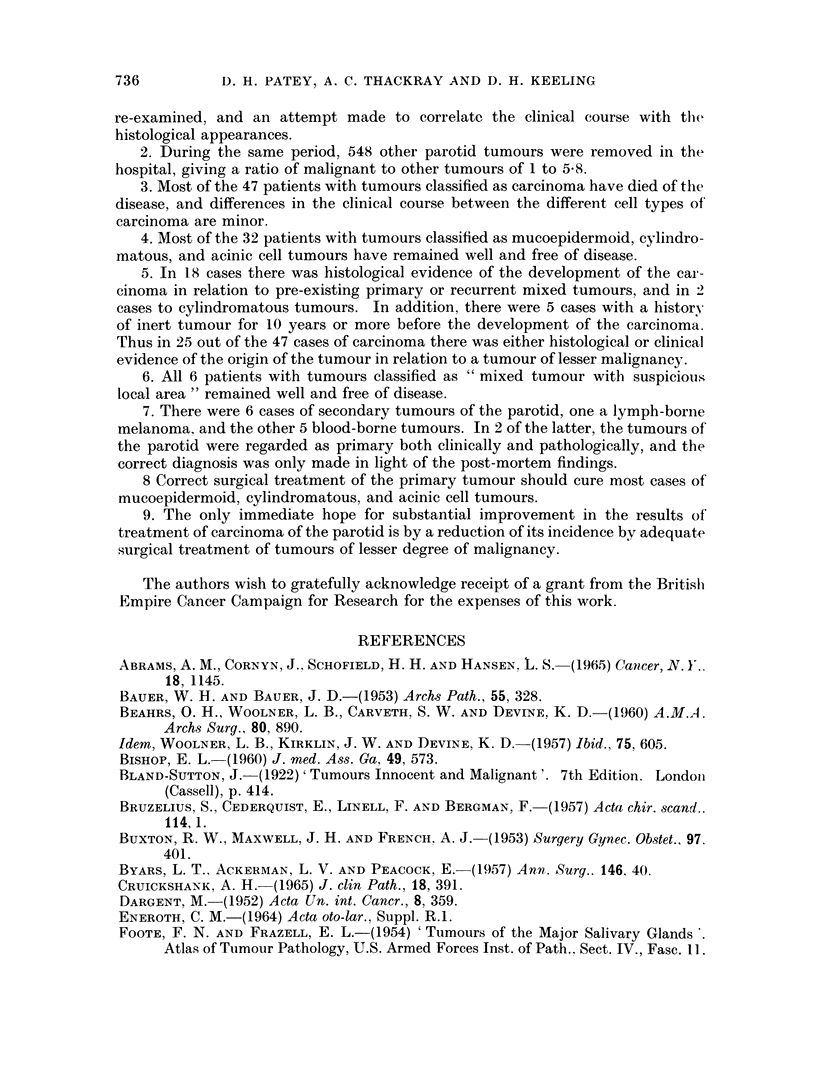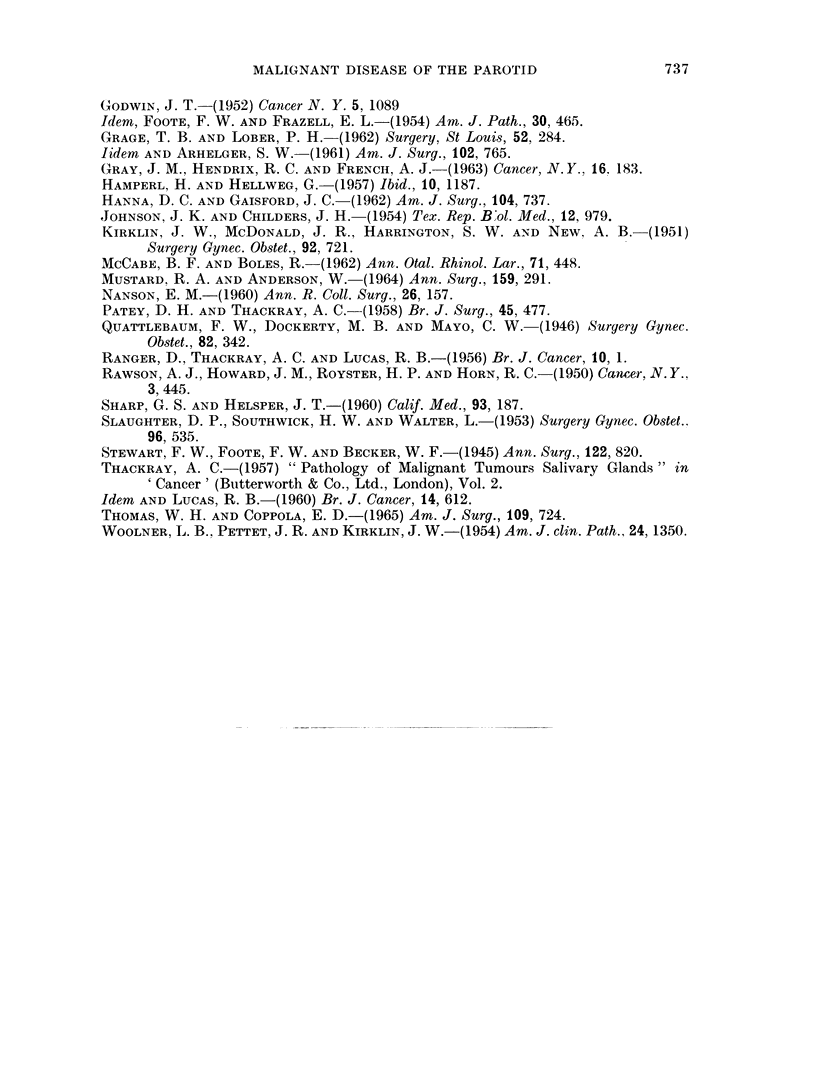# Malignant disease of the parotid.

**DOI:** 10.1038/bjc.1965.84

**Published:** 1965-12

**Authors:** D. H. Patey, A. C. Thackray, D. H. Keeling


### MALIGNANT DISEASE OF THE PAROTID

D. H. PATEY, A. C. THACKRAY AND D. H. KEELING

*From the Department of Surgical Studies and the Bland-Sutton Institute of Pathology, Middlesex Hospital, London, W.1*

Received for publication October 15, 1965

MALIGNANT disease of the parotid is rare, and the prognosis is generally regarded as poor. Treatment is usually by various combinations of surgery and radiotherapy but, as the pertinent published series are mostly recent and the pathological types of tumour varied, it may be difficult to give a prognosis for a particular case and often to decide on the best line of treatment. The conclusions of studies from the earlier half of the present century suffer from the lack of recognition of some now clearly differentiated pathological types, a heterogeneous collection of tumours being grouped together under such headings as " atypical mixed tumours " or " semi-malignant tumours ". Even in some of the more recent papers, though mucoepidermoid, cylindromatous and acinic cell tumours are clearly defined, there is still some terminological confusion owing to the tendency to group them together, particularly as regards results, under the heading of " carcinoma ".

For these reasons, we have thought it worthwhile to record with full documentation the pathological and clinical features, including some long term results, of a series of recently studied cases. We have avoided the term " carcinoma " in connection with the three other types of tumour mentioned above, reserving this term for the more classical cell types of carcinoma.

### MATERIAL AND METHOD OF STUDY

The clinical and pathological records were collected of all patients treated at the Middlesex Hospital between January 1930 and August 1964 in whom a diagnosis of malignant disease of the parotid or of suspected malignant disease of the parotid had been made. In addition, we also reviewed the histological sections of all tumours diagnosed as mixed tumours during the same period and we have included in our study those which we would now diagnose as malignant. All histological sections were re-examined and classified without reference to the clinical histories. The clinical features of the various pathological types were then studied, particularly from the point of view of a possible relation between pathological type and clinical behaviour.

We have previously argued that, because of their tendency to implantation recurrence, mixed tumours should be regarded as of a low degree of malignancy rather than as completely benign like the adenolymphomas (Patey and Thackray, 1958). In the present study, however, we have not included uncomplicated mixed tumours but only tumours of higher degrees of malignancy—cylindromatous, mucoepidermoid, acinic cell tumours, and the various cell types of frank carcinoma, together with a small number of lymphoid tumours.

During the earlier years from which the material is drawn, surgical attempts at cure of malignant disease of the parotid were rarely made, and even biopsy was usually avoided. As a result, since only cases in which histological material was available for study have been included, the rarity of malignant disease of the parotid is exaggerated in the figures from the earlier years. With the development of parotid surgery since the second world war, and almost routine biopsy in cases treated by radiotherapy, the latter years of study include substantially all the cases of the condition treated in the hospital. But here too there is an element of exaggeration, though in the reverse direction, since the hospital has probably in recent years attracted an undue proportion of cases of parotid disease. In spite of these distortions, the fact that the number of established cases of malignant disease of the parotid admitted to a London teaching hospital over 35 years amounts to under 100 probably correctly indicates the rarity of the condition compared with malignant disease in many other regions.

### *Classification*

We will describe later under the respective headings the characteristic histological features of acinic cell, mucoepidermoid and cylindromatous tumours. In many carcinomas, classification according to cell type is difficult on account of mixed appearances, and under these circumstances we have classified them under the predominant type. As will be discussed later, there is now clear evidence that mixed tumours may undergo a change of cell type to become frank carcinomas, and most authors include these under the heading of " malignant mixed tumours ". We have thought it better to classify them under the heading of the cell type of carcinoma which develops.

Based on the histological appearances, the 95 tumours of the series have been subdivided into the following groups:

| | | | | |
|---|---|---|---|---|
| Table | I | Spheroidal cell carcinoma . . . . | 22 | cases |
| ,, | II | Spindle cell carcinoma . . . . | 8 | ,, |
| ,, | III | Adenocarcinoma . . . . . | 11 | ,, |
| ,, | IV | Squamous cell carcinoma . . . . | 6 | ,, |
| ,, | V | Acinic cell tumour . . . . . | 4 | ,, |
| ,, | VI | Muco-epidermoid tumour . . . . | 13 | ,, |
| ,, | VII | Cylindromatous tumour . . . . | 15 | ,, |
| ,, | VIII | Mixed tumour with suspicious local area . | 6 | ,, |
| ,, | IX | Lymphoid tumour . . . . . | 4 | ,, |
| ,, | X | Secondary tumour . . . . . | 6 | ,, |
| | | Total . . . . . . | 95 | ,, |

The main pathological and clinical features of the various groups will now be considered. Figures in brackets refer to the case number detailed in the Tables.

### *Spheroidal Cell Carcinoma* (*Table I*)

### *Pathological findings*

There were 22 tumours classed as spheroidal celled, that is, undifferentiated growths without glandular structures and with the tumour cells of rounded form. In 19 cases an attempt had been made to remove the tumour entire, whilst in the

TABLE I.—*Spheroidal Cell Carcinoma— 22 Cases*

| Case | Year | No. | Sex | Age | History | Treatment | Result |
|---|---|---|---|---|---|---|---|
| 1 | 1933 | 51737 | F. | 64 | Lump 6 months | Local removal + radiotherapy | Died disease 9 years. |
| 2 | 1938 | 4225 | F. | 45 | Lump 1 year | Enucleation—Rupture + radiotherapy | Well 23 years. |
| 3 | 1948 | B66531 | M. | 45 | Recurrent lump following operation 2½ years | Radiotherapy | Died disease 2½ years. |
| 4 | 1948 | B68534 | M. | 60 | Recurrent lump following operation 6 months | Radiotherapy + block dissection | Died disease 5½ years |
| 5 | 1956 | K9099 | M. | 65 | Lump 2 years—4 months rapid growth + facial palsy | Radiotherapy | Died disease 4 years |
| 6 | 1957 | K22943 | F. | 31 | Lump 3 months + facial palsy | Radical parotidectomy + radiotherapy | Well 6½ years. |
| 7 | 1958 | PP4685 | F. | 50 | Recurrence following local removal 4 months | Radical parotidectomy + excision masseter and surrounding tissues + block dissection + radiotherapy | Died disease 1 year. |
| 8 | 1958 | K41209 | F. | 73 | Lump 3 years—6 months rapid growth | Radical parotidectomy | Well 5½ years. |
| 9 | 1960 | PP4922 | F. | 65 | Lump 2 years—recent ulceration | Radical parotidectomy + excision masseter and surrounding tissues | Died disease 6 months. |
| 10 | 1961 | L64986 | M. | 52 | Lump 6 months + lymph nodes | Radical parotidectomy + block dissection | Died disease 1½ years. |
| 11 | 1961 | PP5096 | M. | 65 | Lump 10 years—facial palsy 1 year | Radiotherapy | Died disease 1 year. |
| 12 | 1963 | G14959 | M. | 64 | Lump + pain 1 year | Radical parotidectomy + radiotherapy | Well 1 year. |
| 13 | 1963 | G16386 | F. | 80 | Lump 6 weeks + pain | Radiotherapy | Died disease 6 months. |

*Spheroidal Cell Carcinoma with* Histological *Evidence of Origin in Mixed Tumour*

| Case | Year | No. | Sex | Age | History | Treatment | Result |
|---|---|---|---|---|---|---|---|
| 14 | 1950 | H2505 | F. | 77 | Lump 1 year—recent rapid growth + pain | Radiotherapy | Died disease 10 months. |
| 15 | 1953 | H52565 | M. | 74 | Lump 6 years—6 months rapid growth + pain | Radical parotidectomy + block dissection + radiotherapy | Died disease 2 years. |

TABLE I—*contd.*

*Spheroidal Cell Carcinoma with* Histolegical *Evidence of Origin in Mixed Tumour* (continued)

| Case | Year | No. | Sex | Age | History | Treatment | Result |
|---|---|---|---|---|---|---|---|
| 16 | 1957 | K24545 | M. | 78 | Lump 13 years—3 months rapid growth + pain | Semi-conservative parotidectomy + radiotherapy | Died old age 7 years—no recurrence. |
| 17 | 1958 | K38021 | F. | 78 | Lump 30 years—recent pain + facial palsy | Radical parotidectomy + block dissection + radiotherapy | Died disease 4 years. |
| 18 | 1960 | K66302 | M. | 62 | Recurrence following operation 42 years —3 months rapid growth + facial palsy | Radical parotidectomy | Well 4 years. |
| 19 | 1960 | PP4909 | M. | 34 | Recurrence following operation 15 years—1 year rapid growth | Conservative parotidectomy | Died disease 1 year. |
| 20 | 1962 | K52325 | M. | 34 | Recurrence following operation 7 years—3 years more rapid growth | Semi-conservative parotidectomy | Well 2 years. |
| 21 | 1963 | G24031 | F. | 52 | Recurrence following operation 13 years | Conservative parotidectomy + radiotherapy | Well 3 months. |

*Spheroidal Cell Carcinoma with* Histological *Evidence of Origin in Cylindroma*

| Case | Year | No. | Sex | Age | History | Treatment | Result |
|---|---|---|---|---|---|---|---|
| 22 | 1953 | C84146 | M. | 33 | Lump 1½ years—gradual growth | Multiple operations including radical parotidectomy + excision masseter and surrounding tissues including pinna + block dissection + radiotherapy | Died disease 1 year. |

other 3, biopsies only were available for study, the patients being subsequently treated by radiotherapy. In one of these 3 biopsies (14) there was in fact histological evidence that a mixed parotid type of tumour was also present, as was also the case in 7 of the parotidectomy specimens (15 to 21). The majority of the 8 patients with histological evidence of previous mixed tumour had long clinical histories, in one case going back 42 years (18). These long histories constitute overwhelmingly strong evidence that the carcinoma arose from an existing mixed tumour rather than that the mixed tumour was carcinomatous from the beginning. In 4 of the 8 cases (18, 19, 20 and 21) the carcinomas developed in recurrences of mixed tumours which had been removed 7 to 42 years previously. In several of the specimens the circumscribed area of mixed parotid tumour was hyaline and almost acellular, in marked contrast to the highly active carcinoma apparently developing from it. In one case (22) the presence of a small area of typical well differentiated cylindroma suggested the possible origin of the carcinoma in a cylindroma, though this suggestion is not supported by a long history.

### *Spindle Cell Carcinoma* (*Table II*)

*Pathological findings*

There were 8 carcinomas of the parotid the cells of which were predominantly spindle shaped. In some of these the tumour cells were recognisably of myoepithelial type. Two tumours, histologically very similar, were in young girls and quickly invaded regional lymph nodes and proved fatal (24, 25). Four of the remaining tumours showed histological evidence of origin in pre-existing mixed parotid tumours (26 to 29), the histological evidence being supported by clinical evidence in the form of the previous presence of parotid tumours for periods varying from 8 to 30 years. In 3 of these 4 cases, the carcinoma arose from recurrences developing after surgical removal of the original tumour. In addition, there was 1 case in which there was histological evidence of the origin of the

TABLE II.—*Spindle Cell Carcinoma*—8 *Cases*

| Case | Year | No. | Sex | Age | History | Treatment | Result |
|---|---|---|---|---|---|---|---|
| 23 | 1932 | 39707 | M. | 63 | Lump rapidly growing 1 month | Radiotherapy | Died disease 6 months. |
| 24 | 1953 | H52514 | F. | 9 | Recurrence following operation 6 months—rapid growth | Radical parotidectomy + radiotherapy | Died disease 9 months. |
| 25 | 1961 | K87439 | F. | 13 | Rapidly growing lump—6 months | Radiotherapy + semi-conservative parotidectomy | Died disease $1\frac{1}{2}$ years. |
| *Spindle Cell Carcinoma with* Histological *Evidence of Origin in Mixed Tumour* | | | | | | | |
| 26 | 1942 | A77029 | F. | 38 | Multiple recurrences following original operation 30 years. Traumatic facial palsy | Excision + Radiotherapy | Died disease 7 years. |
| 27 | 1942 | A78905 | F. | 56 | Multiple recurrences following original operation 21 years. Facial weakness | Radiotherapy—repeated for further recurrences | Died disease 10 years. |
| 28 | 1958 | K39606 | M. | 53 | Recurrence following original operation 8 years—recent rapid growth + pain | Radical parotidectomy + block dissection + radiotherapy | Died disease $1\frac{1}{2}$ years. |
| 29 | 1959 | K57524 | F. | 39 | Lump 14 years—recent rapid growth + pain | Radiotherapy | Died disease 1 year. |
| *Spindle Cell Carcinoma with* Histological *Evidence of Origin in Cylindroma* | | | | | | | |
| 30 | 1957 | K19134 | F. | 46 | Lump 21 years—no recent clinical change | Radical parotidectomy | Well $6\frac{1}{2}$ years. |

carcinoma in a cylindroma (30). In this case too, there was clinical confirmation in the form of a history of an inert tumour for 21 years.

### *Adenocarcinoma* (*Table III*)

*Pathological findings*

Sections of the parotidectomy specimen were available for 7 of the 11 cases diagnosed as adenocarcinomas, that is, frankly malignant tumours showing some

TABLE III.—*Adenocarcinoma*—11 *Cases*

| Case | Year | No. | Sex | Age | History | Treatment | Result |
|---|---|---|---|---|---|---|---|
| 31 | 1931 | 12673 | M. | 54 | Lump 15 years—recent rapid growth | Radiotherapy | Died disease 2 months. |
| 32 | 1935 | 74376 | M. | 66 | Lump 6 months | Radiotherapy | Died disease 6 months. |
| 33 | 1945 | B71105 | F. | 67 | Lump 4 years—recent rapid growth + facial palsy | Radiotherapy | Died disease 4 years. |
| 34 | 1949 | B86375 | M. | 54 | Lump 1 month + facial palsy | Radiotherapy + block dissection | Died disease 4 years. |
| 35 | 1953 | H44826 | F. | 47 | Recurrence following operation 11 years— recent rapid growth | Local excision + radiotherapy | Well 11 years. |
| 36 | 1960 | K65846 | F. | 53 | Lump 25 years—recent rapid growth + pain | Semi-conservative parotidectomy | Well 4 years. |
| *Adenocarcinoma with* Histological *Evidence of Origin in Mixed Tumour* | | | | | | | |
| 37. | 1963 | G17165 | F. | 59 | Lump 33 years-recent rapid growth | Radial parotidectomy | Alive with disease 1 year |
| 38 | 1958 | K40254 | M. | 66 | Lump 16 years—no recent change | Conservative parotidectomy | Well 5 years. |
| 39 | 1960 | K65227 | F. | 66 | Lump 6 weeks + facial palsy | Radical parotidectomy + radiotherapy | Died disease 2½ years. |
| 40 | 1961 | K90393 | F. | 73 | Lump 30 years—rapid growth 1 year | Radical parotidectomy + radiotherapy | Alive with disease 2½ years. |
| 41 | 1961 | PP5084 | F. | 76 | Lump " since a girl ". Recent rapid growth. Widespread local disease | Not treated | Untraced—presumed dead. |

tubule formation but without cylindromatous or other specific patterns. In the other four cases biopsies only were taken. In 5 cases (37 to 41) there was clearly recognisable mixed parotid tumour tissue present in addition to the adenocarcinomatous infiltrative part of the growth. The remaining mixed parotid tissue often appeared hyaline and even necrotic in these cases, suggesting that either the carcinoma had arisen in a degenerating mixed tumour or that the onset of malignancy interfered with its nutrition. In a further case there was a history that a mixed parotid tumour had been removed some years before and had recurred, but in the biopsy before radiotherapy there was no recognisable mixed tumour tissue (35). Of the primary adenocarcinomas, one was mucus producing (34) and another had occasional papillary adenocarcinomatous areas (33). Those adenocarcinomas that developed from mixed parotid tumours sometimes showed occasional areas of spindle, spheroidal or even squamous carcinoma.

### *Squamous Cell Carcinoma (Table IV)*

*Pathological findings*

Six tumours in the series were squamous cell carcinomas. Of these, biopsies only were taken in four. In one of the total parotidectomy specimens containing a

TABLE IV.—*Squamous Carcinoma*—6 *Cases*

| Case | Year | No. | Sex | Age | History | Treatment | Result |
|---|---|---|---|---|---|---|---|
| 42 | 1937 | A3092 | M. | 73 | Lump 2 months—rapid growth | Radiotherapy | Died disease few months. |
| 43 | 1942 | A80914 | F. | 47 | Recurrence following original operation 30 years—recent rapid growth + pain | Radiotherapy | Died disease 3 years. |
| 44 | 1955 | H96355 | F. | 73 | Lump 1 month—rapid growth + pain | Radiotherapy | Died disease 4 months. |
| 45 | 1963 | G16839 | M. | 78 | No clinical primary mass lymph nodes neck 2 months | Radiotherapy | Died disease 1 month. |
| 46 | 1963 | G23588 | M. | 68 | Lump 1 year—facial weakness | Radical parotidectomy | Well 6 months. |
| | | | | | *Squamous Carcinoma with* Histological *Evidence of Origin in Mixed tumour* | | |
| 47 | 1959 | K59993 | M. | 77 | Recurrence following original operation 30 years recent rapid growth + plain | Radical parotidectomy + excision masseter and adjacent tissues + block dissection + radiotherapy | Died disease 2 years. |

keratinising squamous carcinoma (47) there was the characteristic outline of a mixed tumour clearly visible when the specimen was cut across, an appearance which was confirmed histologically. As in some previously mentioned cases, much of this mixed tumour was hyaline and degenerate, though still with the structure of the pseudo-cartilagenous areas discernible. Areas of metaplastic squamous epithelium are of course occasionally present in mixed tumours. Another patient (45), from whom the whole parotid was available only at post-mortem, presented with an enlarged hard lymph node in the neck which was removed and found to be almost entirely replaced by keratinising squamous carcinoma. No primary site was found clinically and he was treated by radiotherapy. He died a month later and at the post mortem the parotid was found to show extensive atypical squamous metaplasia of its duct system with atrophy of most of the parenchyma and a small area of invasive carcinoma. This case adds the parotid to the list of sites for latent primary tumour presenting as a secondary enlargment of cervical lymph nodes due to invasion with squamous cell carcinoma.

*Carcinomas (Tables I, II, III and IV)*

*Clinical features*

Our original intention was to attempt to correlate the clinical and histological findings of the different varieties of carcinoma as of the other histological groups. Even a cursory glance at the Tables will show, however, that the clinical findings in carcinoma of the parotid have the same general pattern irrespective of the histological subdivisions. Rather therefore than analysing each type of carcinoma separately, we have considered them together, referring as necessary to any special individual features. With only one or two exceptions, the follow-up is complete and for this we have to thank the efficiency of the Tumour Registry of the Middlesex Hospital. In general we have taken the end of August 1964 as the end point of the study. In the case of patients surviving for several years we have taken the date of the current annual follow-up as the end point. Times of survival are given to the nearest half year except when death has occurred sooner.

*Sex and age.*—Twenty-two of the 47 patients were males and 25 females. Both sexes are thus equally liable to the disease. It is more likely to develop in the later years of life, the average age of the patients on presenting being 57. The disease may, however, arise much earlier, 2 of the patients being children, one aged 9 (24) and the other aged 13 (25) both with spindle cell tumours, while 6 patients were in their thirties. At the other end of the scale, there were 12 patients aged 70 or over, the oldest being 80. While the numbers are too small for firm conclusions, it may be worth noting that 5 of the 6 patients with squamous carcinoma were aged 68 or over.

*History.*—There are two main modes of presentation of carcinoma of the parotid : the first, as a primary tumour in a gland in which nothing abnormal had been noted previously ; the second, as a change in character of a previously inert tumour which had often been present for many years. Under the first heading, a carcinoma of the parotid may present as a symptomless lump indistinguishable clinically from a mixed tumour. On the other hand, a rapid rate of growth of the tumour, pain, or clinical evidence of infiltration may point to the correct diagnosis from the first. These features too, occurring in a previously inert tumour, suggest the possibility of carcinomatous change. Rapid growth or pain, and usually both, was noted in 27 of the 47 cases. Facial palsy is less frequent as an early symptom, and usually only occurs in advanced disease. It was noted in 11 cases in some degree but in some of these was an expression of previous operative trauma rather than the result of the disease. In one case, however, (6) the patient's first complaint was of facial palsy, and the presence of a small lump was only noted when she consulted her doctor for the palsy. The explanation, as revealed at operation, was that the growth had started close to the stylomastoid foramen, into which it had spread.

There is now abundant evidence for the development of carcinomatous change in a mixed tumour, and there can be no doubt of the existence of this phenomenon. We have classified under separate headings in Tables I–IV the 20 cases of the present series in which we found histological evidence that carcinoma had developed in relation to a tumour of lesser malignancy, in 18 cases a mixed tumour and in 2 cases a cylindroma. In most of these 20 cases, there was a history of the presence of an inert lump in the parotid for many years previously. In addition, there were 5 further cases (11, 31, 35, 36, 43) in which, though we did not find any histological evidence of pre-existing mixed tumour or cylindroma, there was a previous history of a lump in the parotid for 10 years or more. Thus the total number of cases in which there was evidence, either from the history or from the microscopical examination, that the carcinoma had arisen from a tumour of lesser malignancy amounted to 25 out of the total of 47, constituting a majority of all cases of carcinoma. Though again the numbers are too small for firm conclusions, it may be worth noting that adenocarcinomas (Table III) illustrate the phenomenon most strikingly with a long history of previous inert tumour or histological evidence of mixed tumour in 8 out of the total of 11 cases.

If we arbitrarily regard as 50 years the length of history in the woman aged 76 (41) who stated that she had had a lump " since she was a girl ", the average length of history of inert tumour in the 25 cases was 19 years. There was one patient with a history of 42 years, 6 more with a 30 years history, and another 3 with a history of 20 years or more. In 10 of the 25 cases, the carcinoma developed in relation to recurrences of the original tumour, and in several of these multiple operations had been performed over the course of the years.

*Treatment.*—During the early years of the present series, surgery of the parotid was generally limited to enucleation of such tumours as were judged suitable for this treatment, and any case diagnosed as carcinoma was likely to be sent for radiotherapy. From the late 1940's onwards, surgeons became more aggressive in their attitude towards parotid tumours, and radical parotidectomy, i.e. parotidectomy with sacrifice of the facial nerve, became the standard surgical procedure for malignant tumours. Occasionally conservative parotidectomy, i.e. parotidectomy with conservation of the facial nerve, or semiconservative parotidectomy, i.e. parotidectomy with partial conservation of the facial nerve, was feasible. The growth may spread outside the parotid to involve adjacent structures, the masseter from its position obviously being most frequently involved and removed. Other structures, parts of which have occasionally been removed, include the temporal fascia and muscle, the periosteum covering the zygoma, the posterior belly of the digastric, and the sternomastoid. In one case, the external ear and the cartilaginous portion of the external auditory meatus were removed (22). Block dissection of the neck was only performed if there was clinical evidence of lymph node invasion. Post-operative radiotherapy was given in many cases, but not as a routine.

*Results.*—The results of treatment of carcinoma of the parotid are bad, irrespective of the type of treatment and of the mode and origin of the disease. Thirty-one of the 47 patients have already died of the disease, 1 is presumed to have done so, 2 though alive still have disease present, and it is likely that some of the more recently treated cases will also die of the disease. The bad results involve both primary growths and those developing on previous inert tumours, and all four histological types, though possibly the results are worst in spindle and squamous cell carcinomas. Death usually occurs within a few months to a couple of years but occasionally patients may survive for many years before dying of the disease, during which time they usually undergo a succession of surgical and radiological treatments. Thus in this series 22 patients died within $2\frac{1}{2}$ years of presenting, while 8 lived for 4 years or more before dying of the disease including 1 for 9 years and 1 for 10 years.

Coming to individual forms of treatment, all 15 patients treated by radiotherapy alone died of the disease, as also did all 9 patients (4, 7, 10, 15, 17, 22, 28, 34, 47) on whom block dissection of the neck was done, and all 4 patients (7, 9, 22, 47) in whom extensive excision of surrounding tissues was necessary, three of whom also had block dissections. It is obvious, however, that the worst types of case were likely to be treated in these ways. The outlook, however, is not entirely black. Nine patients are alive and clinically free of disease for 2 years or more after treatment, 1 for 2 years (20), 2 for 4 years (18, 36), 2 for 5 years (8, 38), 2 for 6 years (6, 30), 1 for 11 years (35), and 1 for 23 years (2) and, in addition, there is one patient who died of old age but free of disease 7 years after treatment (16). The treatment carried out in these 10 so far successful cases was local excision and radiotherapy in 2 cases (2, 35), semiconservative parotidectomy in 3 cases (16, 20, 36), in one of which radiotherapy was also given, conservative parotidectomy in 1 case (38), and radical parotidectomy in 4 cases (6, 8, 18, 30) in 1 of which radiotherapy was also given (6). The fact that 6 of these 10 successful cases had operations less than radical parotidectomy indicates that these growths were certainly more localised and probably also less malignant. The case of the patient that has survived 23 years (2) is particularly interesting, since the operation was an attempted enucleation during which the tumour burst, the operation being followed by radiotherapy. In the light of this surprising result, we have critically re-examined the microscopical sections from this case, but although one observer has suggested that this is in fact a poorly differentiated acinic cell tumour we found no features to substantiate this. The separation of the less well differentiated examples of special types of tumour from the general group of carcinomas is difficult, but may obviously be important if there are significant differences of behaviour.

### *Acinic Cell Tumour (Table V)*

### *Pathological findings*

Acinic cell tumours, though known for many years, have only been clearly delineated as an important group of malignant salivary neoplasms in the last twelve years (Buxton *et al.*, 1953 ; Godwin *et al.*, 1954). The great majority occur in the parotid gland (Abrams *et al.*, 1965).

The four cases of acinic cell tumour in the present series were all originally diagnosed, both clinically and histologically, as either mixed tumours or adenomas. Three of the 4 cases presented as recurrent tumours.

Abrams *et al.* (1965) noted four tissue patterns in their 77 acinic cell tumours. The most frequent was a solid parenchymatous form, and a microcystic configuration was extremely common, at least in some degree. Papillary cystic and follicular growth patterns were also seen. Histologically the operation specimens from our cases showed typical well-differentiated acinic cell tumours, the cells of which were regular in size and staining and resembled normal parotid acinic cells.

### *Clinical features*

*Sex and age.*—All four patients in our series were women, and this is in accordance with the general experience that women predominate in this group. One patient was aged 39 but the other 3 were over 60, 2 being over 70. In all cases, however, the condition had started at least 8 years previously.

TABLE V.—*Acinic Cell Tumour*—4 Cases

| Case | Year | No. | Sex | Age | History | Treatment | Result |
|---|---|---|---|---|---|---|---|
| 48 | 1931 | 17398 | F. | 61 | Recurrence following operations 11 years + 4 years | Enucleation + curettage | Further recurrence and operation 11 years. Died 12 years other causes. |
| 49 | 1943 | A92856 | F. | 77 | 10 years gradual growth | Radiotherapy + excision | Died age 99—no recurrence. |
| 50 | 1952 | H31691 | F. | 73 | Recurrence following operation 8 years. Traumatic facial palsy | Radical parotidectomy | Died 7 years—other causes. |
| 51 | 1962 | G8950 | F. | 39 | Recurrence following operation 8 years—further recurrence 5 years + 1 year. Pain feature from beginning | Radical parotidectomy + excision masseter and adjacent tissues | Well 2 years. |

*History.*—Only 1 patient (49) presented with a primary tumour, and a history of a slow growing tumour without any special clinical features. The other 3 cases presented with local recurrences and a history in 2 cases of multiple operations. In 1 case (51) pain was a striking feature both with the primary tumour and with the recurrences, suggesting the formation of a pain producing agent by the tumour cells. Grage and Lober (1962) also noted distressing pain as being present in 2 of their 8 cases.

*Results.*—Our cases are too few to allow firm conclusions but the tentative picture is of a tumour of low malignancy with a marked tendency to recur following limited local excision. In 2 of the 4 cases sacrifice of the facial nerve was necessary but no case showed lymph or blood borne metastases, and the results as judged by survival were good.

*Mucoepidermoid Tumour* (*Table VI*)

*Pathological findings*

Mucoepidermoid tumours have only recently been segregated (Stewart, Foote and Becker, 1945) and were at first divided into benign and malignant types. At present, however, the tendency is to regard them all as malignant though in different degree.

Sections of the surgically removed tumours of all the 13 patients in this group were available for study, though in four cases the operation at the Middlesex Hospital was for removal of recurrent growth. The tumours varied in size from $\frac{3}{4}$ in. to $1\frac{1}{2}$ in. in their greatest dimension. On cutting across the specimen they were described as ill-defined collections of mucus filled cysts in about half the cases, and as solid apparently circumscribed tumours in the others. One case was described as a firm circumscribed tumour 1 in. diameter containing cystic spaces up to $\frac{3}{16}$ in.

Microscopically, all contained a characteristic mixture of epidermoid and mucus secreting neoplastic tissues, though in varying proportions. One case had the two

TABLE VI.—*Mucoepidermoid Tumour*—13 *Cases*

| Case | Year | No. | Sex | Age | History | Treatment | Result |
|---|---|---|---|---|---|---|---|
| 52 | 1953 | H53515 | F. | 39 | Lump 1 year | Conservative parotidectomy + excision masseter + radiotherapy | Well 10½ years. |
| 53 | 1953 | H52271 | M. | 16 | Lump 1 year | Conservative parotidectomy + excision of recurrence 1 year + radiotherapy | Well 11 years. |
| 54. | 1954 | H66521 | F. | 42 | Lump " many years " | Local excision + radiotherapy | Well 10 years. |
| 55 | 1954 | H79309 | M. | 30 | Lump 3 months | Conservative parotidectomy | Well 10 years. |
| 56 | 1956 | J25670 | M. | 60 | Lump 2 years—recent increase in size + pain | Semi-conservative parotidectomy + radiotherapy | Died disease 6 years. |
| 57 | 1956 | K4933 | M. | 62 | Lump 1 year | Semi-conservative parotidectomy | Well 8 years. |
| 58 | 1957 | PP4588 | F. | 57 | Recurrence following operation 2 years | Conservative parotidectomy + block dissection + 2 operations for further recurrences + radiotherapy | Well 7 years. |
| 59 | 1958 | PP4639 | F. | 60 | Lump " some years "—recent increase in size | Conservative parotidectomy | Died myocardial infarction 4 years—no recurrence. |
| 60 | 1959 | K54184 | M. | 53 | Lump 1½ years + pain and facial palsy | Radical parotidectomy + radiotherapy | Died myocardial infarction 2½ years—no recurrence. |
| 61 | 1959 | K55421 | M. | 35 | Recurrence following operation 1 year | Conservative parotidectomy | Well 4 years. |
| 62 | 1960 | K43086 | F. | 75 | Lump 4 months | Conservative parotidectomy | Well 4 years. |
| 63 | 1960 | PP4921 | M. | 52 | Recurrence following operation 4 months | Radical parotidectomy + block dissection + radiotherapy | Not traced. |
| 64 | 1962 | PP5163 | F. | 39 | Recurrence following operation 3 months | Semi-conservative conservative parotidectomy + radiotherapy | Well 2 years. |

equally represented, six were predominantly mucoid and cystic, six epidermoid. Ten cases were considered to be of low grade malignancy, and three of high. One of the latter was the only one showing keratinisation, the epidermoid elements in all the others taking the form of sheets of polyhedral cells often with rather clear cytoplasm. In some tumours, occasional goblet cells were scattered among the epidermoid cells, but most often the mucoid cells were concentrated in groups, usually around cysts. In some of the more cystic tumours, papillary structures covered by mucus secreting cells projected into the cysts. In these more mucoid tumours, there were varying degrees of extravasation of mucus into the interstices of the gland, and it appeared that these gave rise to a reaction eventually resulting in considerable fibrosis. There were invaded lymph nodes in three cases ; one patient (58) who had a block dissection four years after the second recurrence of her tumour had a muco-epidermoid tumour of the most regular and low-grade appearance.

*Clinical features*

*Sex and age.*—Seven patients were males and 6 females. There was a wide scatter of ages from 16 to 75 with 4 patients in their thirties. The average at 48 was slightly less than that of carcinoma and of cylindromatous tumours.

*History.*—Six patients presented with primary tumours without any special additional clinical features. Two gave a history of more recent rapid growth, in one case with associated pain ; in addition there was one patient who presented with a painful lump with associated facial palsy. The history of rapid growth and pain is thus appreciably less frequent in muco-epidermoid tumour than in carcinoma and cylindroma. Four presented with recurrences following recent local operations for what were thought to be mixed tumours.

*Treatment.*—All patients were treated primarily by surgery. Radiotherapy was given as an ancillary treatment in 8 cases, and there was also one patient in whom, before he came under our care, radiotherapy had caused a marked but temporary reduction in size of the growth and improvement of the facial paralysis (60). In 7 patients the operation performed was conservative parotidectomy, and in 3 of these infiltrated surrounding tissues were removed either at the primary operation (52), or in 2 cases (53, 58) at operations for recurrence. Three patients were treated by semiconservative parotidectomy (56, 57, 64) 2 by radical parotidectomy (60, 63), and the remaining patient by local excision (54). We performed a block dissection for invaded lymph nodes in 2 patients (58, 63), and in a third (53) there was microscopical invasion of macroscopically normal jugulo-digastric lymph nodes which we had merely removed locally for routine biopsy.

*Results.*—The results of treatment in this group were good. Only one patient is known to have died of his disease—6 years after operation (56). In addition, one patient (63) returned home to Pakistan after operation and has not been traced since. Nine patients are alive and well at 2 years, 4 years (2 cases), 7 years, 8 years, 10 years (3 cases) and 11 years. Two died of other causes without recurrence of the disease, 1 at 2 years and the other at 4 years after operation. The good response to treatment of muco-epidermoid tumours is also brought out by two other observations. All 3 patients on whom excision of infiltrated surrounding tissues was necessary are alive and well, and 2 of the 3 patients with invaded lymph nodes are alive and well. The third was the patient from Pakistan on whom we performed a block dissection, and whom we have not since traced.

We attribute the good results in part to the radiosensitivity of muco-epidermoid tumours. This is illustrated by our experience in case 53, the patient with the microscopically invaded jugulo-digastric lymph nodes. Our original operation of conservative parotidectomywas followed by an infiltrating recurrence at the upper attachment of the sternomastoid. At the second operation we felt convinced that excision was incomplete, and he was given post-operative radiotherapy. The patient is alive and well 11 years later.

*Cylindromatous Tumour (Table VII)*

*Pathological findings*

Both typical mixed parotid tumours and cylindromatous tumours are made up of both the cell types of the normal parotid ducts. In the mixed parotid tumour, the myoepithelial cells are separated out by mucoid material to give the characteristic cartilage-like appearance, whereas in the cylindroma the myoepithelial cells remain in compact groups surrounded by cylindrical sheaths of material which is typically hyaline but may be mucoid. The most important distinction between the two types of tumour, however, is that the cylindromatous salivary gland tumour is infiltrative in its growth, in contrast to the mixed tumour which tends to remain circumscribed. Areas in otherwise typical mixed tumours may show the cylindromatous pattern, but this is generally agreed not to alter the behaviour of the growth in which they are found (Eneroth, 1964). The typical cylindromatous histological pattern is subject to variations, many of which have been described elsewhere (Thackray and Lucas, 1960). In particular, they have a tendency after years of slow infiltrative growth to take on frankly malignant characteristics with rapid spread and metastasis, the metastases often not at first looking cylindromatous. Occasionally the typical cylindromatous growth may metastasise as such, and patients may live for some time in apparent health with secondaries of this type in their lungs.

There were 15 tumours in this group in the series. The operation specimens of 9 cases were from the primary removal, two (72, 73) after histories of a lump being present for 20 years and one (70) for 14 years, all these last three having shown recent more rapid growth. The other patients had operations for tumours previously biopsied elsewhere, for recurrent tumours—in one case (78) there had been four recurrences over a period of 34 years—or biopsies only followed by radiotherapy. The tumours, with two exceptions, were firm white with rather indefinite infiltrative edges and usually between 1 in. and 2 in. across. One was described as multinodular and two were apparently circumscribed. One of the latter (65) had been shelled out as a mixed parotid tumour and following radiotherapy gave no further trouble during the 7 years of follow-up. The other apparently circumscribed tumour (69) was very cellular and in places the cylindromatous pattern was hard to make out. Microscopically, 9 had the typical cylindromatous pattern, 6 hyaline and 3 mucoid; the others showed some variation. In one case (74) there was the pseudo-neurinomatous pattern referred to elsewhere (Thackray and Lucas. 1960), whilst a markedly cellular tumour has been mentioned above. The microscopic appearance of the tumour edge was nearly always infiltrative with islands of tumour cells beyond the apparent naked-eye limits of the tumour. The lateness or absence of distant metastases in these tumours left time for extensive and sometimes repeated plastic operations (66). There was no case with

TABLE VII.—*Cylindromatous Tumour*—15 *Cases*

| Case | Year | No. | Sex | Age | History | Treatment | Result |
|---|---|---|---|---|---|---|---|
| 65 | 1940 | A55555 | F. | 50 | Lump 5 years—recent growth | Enucleation + radiotherapy | Well 7 years —untraced since. |
| 66 | 1945 | PP1981 | F. | 42 | Lump 9 years—recent growth + pain | Radiotherapy + multiple operations | Died disease 14 years. |
| 67 | 1948 | B60494 | M. | 61 | Lump 1½ years | Radiotherapy | Well 15 years. |
| 68 | 1954 | H71881 | M. | 72 | Recurrence following operation 2 years | Radiotherapy | Died disease 6 months. |
| 69 | 1955 | C96384 | F. | 48 | Lump 9 months—growing | Conservative parotidectomy | Well 8 years. |
| 70 | 1956 | J4216 | F. | 58 | Lump 14 years—recent growth + pain | Semi-conservative conservative parotidectomy | Died 4 years—carcinoma cervix. |
| 71 | 1957 | K19678 | F. | 31 | Recurrence following operation 2 years | Conservative parotidectomy excision masseters and temporals + radiotherapy | Well 7 years. |
| 72 | 1957 | PP4484 | M. | 45 | Lump 20 years—recent growth + pain | Conservative parotidectomy + radiotherapy | Well 7 years. |
| 73 | 1958 | K41972 | F. | 61 | Lump 20 years—recent growth + pain | Conservative parotidectomy + radiotherapy | Well 5½ years. |
| 74 | 1959 | K48001 | F. | 30 | Recurrence following operation 2 years | Conservative parotidectomy | Well 3 years—untraced since. |
| 75 | 1960 | K69006 | F. | 68 | Lump 1½ years—growing + facial weakness | Radical parotidectomy + excision masseter and surrounding tissue + radiotherapy | Well 3 years. |
| 76 | 1963 | G20491 | M. | 50 | Lump 5 years | Conservative parotidectomy | Well 1½ years. |
| 77 | 1964 | G26492 | F. | 53 | 6 months pain + intermittent swelling | Conservative parotidectomy + radiotherapy | Still under treatment. |
| 78 | 1964 | G26439 | F. | 59 | Multiple recurrences and operations following lump 34 years | Radiotherapy | Still under treatment. |
| 79 | 1964 | G34309 | M. | 42 | Operation for intermittent pain and swelling 4 years —diffuse recurrent infiltrating mass with proptosis + ophthalmoplegia. Traumatic facial palsy | Radiotherapy | Still under treatment. |

clinical evidence of lymph node invasion and in the few cases in which lymph nodes were removed for biopsy histological examination showed no growth.

*Clinical features*

*Sex and age*.—Five of the 15 patients were males and 10 females. Like carcinoma, the disease is more likely to present in the later years of life, though the average age of 51 is a little lower than that of carcinoma. There were no cases of cylindromatous tumours in children.

*History*.—Like carcinomas, cylindromatous tumours may present as symptomless lumps in the parotid without any special features clinically to distinguish them from other tumours ; they may also present as lumps the rapid increase in size of which, sometimes associated with pain, arouses the suspicion of malignancy. At times as with carcinoma, this active phase is superimposed on a long history of an inert lump—as already noted, in 2 cases of this series for 20 years. In contradistinction to carcinoma, however, in none of our cases did we find histological evidence of the cylindromatous tumour having been superimposed on a mixed tumour. While it is possible, as in some carcinomas, that the tumour had destroyed all evidence of the mixed tumour from which it arose, this is unlikely to have happened in every case. Until histological evidence to the contrary is produced, our experience would lead us to regard cylindromatous tumours of the parotid as occuring in two forms ; a clinically inert form which may persist unchanged for years ; and a clinically active form which may either arise de novo or be superimposed on the inert form. Facial palsy was present in two patients (75, 79), but in one it was at any rate in part traumatic from previous surgery. This last patient had in addition partial ophthalmoplegia from involvement of the orbit. One patient presented with a history of recurrent pain and swelling of the parotid, which led to a diagnosis of recurrent parotitis (77); the diagnosis of cylindromatous tumour was only made on histological examination of the parotidectomy specimen.

*Treatment*.—Radiotherapy was given either alone or ancillary to surgery in 11 of the 15 cases. One patient (67) is alive and apparently free of growth 15 years after radiotherapy for a tumour for which the only surgical procedure was biopsy. Conservative parotidectomy, which was performed in 7 cases, was the surgical procedure most often employed, in one case (71) together with extensive excision of infiltrated surrounding tissues ; in addition there was one case in which semi-conservative parotidectomy was performed. Radical parotidectomy was performed only once. Block dissection of the neck was not called for in any case of the tumour in this series.

*Results*.—The results of treatment of cylindromatous tumours of the parotid are much better than those of carcinoma. Thus, excluding the 3 recent ones still under treatment, 7 of the remaining 12 patients are still alive and well at 1½ years (76), 3 years (75), 5½ years (73), 7 years (71, 72), 8 years (69) and 15 years (67) after treatment ; 2 were well when last seen before being lost to follow up at 3 years (74) and 7 years (65) ; and a 10th patient was free of parotid disease when she died of another cause at 4 years (70). On the other hand, 2 patients (66, 68) have died of the disease, one (66) after 14 years of multiple operations and multiple courses of radiotherapy for recurrent extensions of the disease, a story well known in cylindromas of the minor salivary glands (Ranger, Thackray and Lucas, 1956). Another index of the better prognosis of cylindromas as compared with that of carcinomas is provided by cases 71 and 75, in which the patients are still well and apparently free of disease at 7 years and 3 years in spite of wide excision of involved tissues around the parotid being necessary. As already noted, all cases of carcinoma in which this procedure was necessary died of the disease.

### *Mixed Tumour with Suspicious Local Area* (*Table VIII*)

*Pathological findings*

During the period covered by this study there were six patients whose tumours were reported by the pathologist to contain areas suggestive of malignancy in

TABLE VIII.—*Suspicious Local Area in Mixed Tumour*—6 *Cases*

| Case | Year | No. | Sex | Age | History | Treatment | Result |
|---|---|---|---|---|---|---|---|
| 80 | 1941 | A66243 | F. | 67 | Lump 3 years | Enucleation + radiotherapy | Died accident 11 years. |
| 81 | 1949 | B16515 | M. | 54 | Recurrence following operation 4 years | Local removal + radiotherapy | Well 12 years. |
| 82 | 1950 | H156 | M. | 57 | Lump 1½ years — growing | Parotidectomy + radiotherapy | Well 13 years. |
| 83 | 1953 | C86657 | F. | 55 | Lump 37 years— 2 years growing | Enucleation | Well 10 years. |
| 84 | 1961 | K83412 | M. | 51 | Lump? length of history | Enucleation + radiotherapy | Well 2 years. |
| 85 | 1963 | G16918 | F. | 63 | Multiple operations + radiotherapy over 46 years— Traumatic facial paralysis | Radical parotidectomy + excision masseter, temporalis and other involved tissues + plastic repair | Well 1½ years. |

otherwise typical mixed parotid tumours. It would be very difficult and of doubtful value to try to specify or illustrate the degree of cellularity or cellular atypicality which prompted this diagnosis, but it will be seen that these six form a very small proportion of all cases of mixed parotid tumour seen during the period. Four of them had had primary mixed tumours removed, three by enucleation, and three were given post-operative radio-therapy on the strength of the reported suspicion. The other two cases in the group (81, 85) had recurrent tumours removed which showed areas of marked cellularity, with mitotic activity in one of them. Case 85 is of interest in that her history went back 46 years. The most recent operation—she had had two or three attempts at removal and radiotherapy in the interval—showed one of the numerous nodules present to be solid and compact. All six cases had been diagnosed clinically as mixed tumours and operated on with this diagnosis in mind and the macroscopic appearance of the specimen was consistent with this ; it was only when the sections were examined that the suspicion of malignancy arose. There has been no recurrence or metastasis in any of these six cases since the Middlesex operations.

### *Lymphoid Tumours* (*Table IX*)

The four lymphoid tumours were all in women, 3 of whom were over 60. The tumours appeared to fall into 2 groups. Two (88, 89) were localised lesions in an otherwise normal parotid gland, and had apparently arisen in the lymph nodes normally present within the anatomical limits of the gland. Both these patients had their tumours removed surgically, and following reports of lymphosarcoma and reticulosarcoma respectively were treated with radiotherapy. Both are still alive, though case 88 has had radiotherapy to further tumour in the orbit, and case 89 has developed splenic enlargement and axillary lymph node involvement.

TABLE IX.—*Lymphoid Tumour*—4 *Cases*

| Case | Year | No. | Sex | Age | History | Treatment | Result |
|---|---|---|---|---|---|---|---|
| 86 | 1956 | K12068 | F. | 46 | Lump 6 years—recent increase in size and facial weakness | Radical parotidectomy—no radiotherapy "malignant lymphoma" | Died 8 years ? cause. (well 7 years) |
| 87 | 1960 | K66772 | F. | 66 | Lump 3 months | Radical parotidectomy + radiotherapy "lymphosarcoma" | Died 2 years other cause. |
| 88 | 1961 | K78050 | F. | 63 | Lump 9 months | Conservative parotidectomy + radiotherapy + further radiotherapy for recurrence. "lymphosarcoma" | Well 3 years. |
| 89 | 1962 | K50136 | F. | 73 | Lump 6 months | Local excision + radiotherapy "Reticulosarcoma" | No local recurrence 2 years. Enlarged Spleen and axillary lymph nodes |

The other 2 cases (86, 87) were different in that the glands showed diffuse changes of chronic parotitis. In this condition, localised tumour-like swellings may develop which, if isolated in an otherwise relatively normal gland, are sometimes known as "benign lymphoepithelial lesions" (Godwin, 1952). There are characteristic solid epithelial structures in a lymphoid stroma, but the latter may be very hyperplastic and raise suspicions of malignancy. In these two cases, the diagnosis of malignancy of the lymphoid element was made histologically. In both cases radical parotidectomy was performed. In the first (86) there was no recurrence at a 7 year follow up, but the patient died 8 years after operation, the cause not being ascertained. The second patient (87) showed no sign of further trouble up till her death from other causes 2 years after operation.

These lymphoid tumours constitute a difficult group, and it is of interest that Foote and Frazell (1954) were unable to find a single case of primary malignant lymphoma in their very large series of parotid tumours. Grage and Lober (1962)

on the other hand report 3 cases diagnosed as malignant lymphomas in which the patients were alive and well at 7, 8 and 11 years after operation. It would therefore seem that long survival after treatment of primary malignant lymphoma of the parotid is possible.

### *Secondary Tumours* (*Table X*)

The parotid and its included lymph nodes are rarely the site of metastatic tumours. In most large series of parotid tumours, however, one or more examples

TABLE X.—*Secondary Tumours*—6 *Cases*

(1) *Blood borne*

| Case | Year | No. | Sex | Age | History | Treatment | Result |
|---|---|---|---|---|---|---|---|
| 90 | 1941 | A63305 | F. | 63 | Lump 1 year—pulsating—no general symptoms | Radiotherapy + excision Diagnosed—angioendothelioma | Died 4½ years—carcinoma kidney. |
| 91 | 1952 | C38087 | M. | 45 | Lump 3 months—ill man | Radiotherapy | Died 2 months—carcinoma lung. |
| 92 | 1955 | H96881 | M. | 86 | Lump? length of history—ill man | Radiotherapy | Died 2 months carcinoma pancreas. |
| 93 | 1959 | K52116 | F. | 65 | Lump 8 weeks— no general syptoms | Radiotherapy—excellent immediate response | Died 2½ years carcinoma stomach. |
| 94 | 1961 | K92448 | M. | 59 | Lump 5 months—ill man | Nil | Died 1 week—carcinoma lung. |
| (2) *Lymph borne* | | | | | | | |
| 95 | 1962 | G8400 | M. | 41 | Lump 4 months—operation for melanoma temple 2 years previously. | Conservative parotidectomy | Well 2 years—melanoma in lymph node. |

of secondary melanoma limited to the intraparotid lymph nodes will be found, the primary site as in our one case (95) being on some adjacent area of skin. The clinical appearances do not allow the differentiation from a primary tumour to be made with certainty. In our one case, conservative parotidectomy was possible and the patient was alive and well at 2 years.

In the other 5 cases, the deposits in the parotid were blood borne from the lung—2 cases (91, 94), pancreas (92), stomach (93) and kidney (90). The 2 patients with carcinoma of the lung and the one patient with carcinoma of the pancreas were obviously very ill when they first presented and no biopsy of the parotid lumps was performed. On the other hand, the woman of 63 with the secondary in the parotid from a latent primary in the kidney had had the lump for a year, and the fact that it pulsated perhaps should have led to the correct diagnosis being considered. Even after removal, the correct diagnosis was not made histologically though, on review of the section in light of the information that the patient had died four years after operation from a renal carcinoma, the true nature of the parotid growth was evident. In the case of carcinoma of the stomach too, it was only appreciated that the growth in the parotid was not a primary on a review of the histological section knowing the post-mortem findings.

### DISCUSSION

In general our findings are similar to those reported in other published papers. Allowing for slight differences of material our total of 89 primary malignant tumours of the parotid collected in just under 35 years is comparable with the 74 cases of Mustard and Anderson in 25 years from the Toronto General Hospital and with the 68 cases of malignant disease of the major salivary glands reported by Grage and Lober in 25 years at the University of Minnesota Hospitals. The condition is thus a comparatively rare type of malignant neoplasm. However, the figures of any series of verified tumours earlier than the last two decades must under-estimate the frequency of the condition owing, in the earlier years, to the infrequency of surgical attack, and consequent lack of material for histological examinations. Table XI, which gives some details of some of the larger series published since 1950, shows a fair degree of agreement in the histological distribution of primary epithelial malignancies. For most of the authors quoted figures are also available showing malignant neoplasms as a percentage of all parotid tumours ; in most series the authors find between a quarter and a third of all parotid neoplasms malignant. During the 35 years of the present series a total of 463 mixed parotid tumours were operated on at the Middlesex Hospital together with 85 benign tumours. Our figure of 15 per cent as the proportion of malignant to total parotid tumours is in close agreement with the figures of the recent series of Eneroth (1964) and of McCabe and Boles (1962).

There is general agreement in the literature that carcinoma may develop from mixed tumours, usually after the lapse of many years, and it is of interest and indeed of importance to assess the incidence of malignancy in mixed tumours. The mixed tumours may be either long-standing primary tumours or recurrences following unsuccessful surgery. Dargent (1952) found no evidence that previous

*Table XI—Distribution of Primary Epithelial Malignancies in the Parotid, and Percentage of Parotid Tumours Malignant, after Various Authors.*

| Author | Year | Total Number | Carcinomas | Acinic Cell Tumours | Cylindromatous Tumours | Mucoepidermoid tumours | Percentage malignant |
|---|---|---|---|---|---|---|---|
| | | | % | % | % | % | % |
| Rawson *et al.* | 1950 | 32 | 50 | — | 13 | 37 | 26 |
| Kirklin *et al.* | 1951 | 151 | 65 | — | 22 | 13 | 22 |
| Bauer and Bauer | 1953 | 13 | 31 | — | 31 | 38 | 11 |
| Foote and Frazell | 1954 | 261 | 51 | 8 | 6 | 35 | 34 |
| Johnson and Childers | 1954 | 45 | 67 | 4 | 2 | 27 | 30 |
| Bruzelius *et al.* | 1957 | 49 | 57 | 8 | 6 | 29 | 27 |
| Byars *et al.* | 1957 | 114 | 69 | — | 15 | 16 | — |
| (children) | | (6) | (80) | | | (20) | (26) |
| Nanson | 1960 | 33 | 85 | — | — | 15 | 31 |
| Sharp and Helsper | 1960 | 38 | 50 | 11 | 18 | 21 | 23 |
| Hanna and Gaisford | 1962 | 68 | 50 | 10 | 6 | 34 | 26 |
| McCabe and Boles | 1962 | 86 | 51 | 16 | 30 | 3 | 15 |
| Grage and Lober | 1962 | 47 | 32 | 17 | 19 | 32 | — |
| Mustard and Anderson | 1964 | 74 | 44 | 14 | 14 | 28 | 26 |
| Eneroth | 1964 | 119 | 25 | 30 | 16 | 29 | 15 |
| Present authors | 1965 | 85 | 61 | 5 | 19 | 15 | 15 |

surgical intervention showed any correlation with the subsequent development of carcinoma. Following Foote and Frazell most authors prefer to classify these cases as " malignant mixed tumours ". Since malignancy depends on the carcinoma rather than on the mixed tumour a classification under the heading of the carcinoma seems more rational particularly as they seem to behave in the same way as carcinomas developing without previous tumour. Histologically the carcinomas may be adenocarcinomas, squamous cell carcinomas, undifferentiated spheroidal or spindle cell growths. The comprehensive term " malignant mixed tumour " while being a clinical convenience is therefore pathologically unhelpful. As already noted, out of our 47 cases of carcinoma 25 showed evidence of origin in a tumour of lower malignancy. If we ignore the 2 cases with histological evidence of origin in a cylindroma, and accept the 5 cases with clinical evidence only as mixed tumours, we have 23 carcinomas arising from mixed tumours. This gives an incidence of approximately 5 per cent of malignant change in mixed tumours. This is higher than the estimate of 2 per cent of Rawson *et al.* (1950) and the 3 per cent estimated earlier by one of the present authors (Thackray, 1957). Beahrs *et al.* (1957) reviewing cases seen at the Mayo Clinic stated that 29 of 178 carcinomas showed histological evidence of transformation from mixed tumours ; of these 21 were dead in less than 6 years and 2 were alive but with recurrent disease. Slaughter *et al.* (1953) found that 12 of 55 cases of carcinoma originated in mixed tumours. We have not found any report of frank carcinoma developing from cylindromatous tumours corresponding with the two cases of our series. Neither in our series nor in the literature have we found evidence of carcinoma arising from mucoepidermoid tumours though both Grage and Lober (1962) and Foote and Frazell (1954) speculate that cases of squamous cell carcinoma with a long history of an inert tumour may have arisen in this way. Rawson *et al.* (1950) classify their series like us. They consider that " the adenocarcinomas, epidermoid carcinomas and undifferentiated carcinomas may be grouped together as highly malignant tumours ", and grouped these under the general heading of carcinoma. This is in close agreement with our findings which confirmed this high degree of malignancy, with most patients dying of their disease irrespective of the treatment adopted. Beahrs *et al.* (1960) adopted a somewhat similar classification.

We have not felt the justification for further subdivision of *adenocarcinomas* into papillary growths, mucus secreting tumours and solid or trabecular adenocarcinomas, as total numbers were not large, and no useful information was given by any such attempt. Our experience is broadly similar to that of others ; these are highly malignant tumours.

The undifferentiated *spheroidal celled carcinomas* behaved as expected from the histological appearances, as high grade malignant neoplasms. Over one-third of these cases were apparently superimposed on previous mixed tumours, often of very long standing. However, the behaviour and prognosis of these tumours does not appear to be influenced by their origin—the course followed is the same as that found in carcinomas arising *de novo.* In addition there was one tumour that may have arisen from a cylindroma.

The small group of tumours that were predominantly *spindle cell* in type were highly malignant. The existence of this group has not been stressed previously. Kirklin *et al.* (1951) found a small number of spindle cell tumours which failed to show evidence of collagen production, pursued a very malignant course, and were classified as sarcomas. These tumours may well correspond to our spindle cell group of carcinoma. Bishop (1960) felt that " most so-called sarcomas of salivary glands prove to be atypical carcinomas, metastases, or invasion from without ".

There are remarkable discrepancies in the reported frequency of *squamous cell carcinoma* possibly due in part to the histological borderline accepted between them and high grade mucoepidermoid neoplasms. The problem of mucus in squamous cell carcinomas and the work of Hamperl and Hellweg (1957) is discussed by Gray *et al.* (1963) in this context. Our frequency of 7% of primary epithelial malignancies is in good agreement with three American series. Kirklin *et al* (1951) noted 8% in the Mayo Clinic series, 9% was reported by Sharp and Helsper (1960) and Foote and Frazell (1954) quote 10%. These figures contrast with the recent Scandinavian series of Eneroth (1964) and Bruzelius *et al.* (1957) who found only one case between them. The cases in the present series grouped as squamous cell carcinomas show an extremely poor prognosis, in agreement with the findings of Hanna and Gaisford (1962), and Foote and Frazell (1954) : these latter authors noted that their 10% of squamous cell carcinomas were high grade tumours both histologically and clinically. Several authors agree that these tumours occur in an older age group, are commoner in males, and have in general a short history.

On the treatment of developed carcinomas and the possibility of improving results, there is a difference of opinion. Grage and Lober strongly advocate " more aggressive surgical treatment ", while Mustard and Anderson take an opposite view. They write : " of ten patients subjected to radical total parotidectomy (i.e. with sacrifice of the facial nerve) not one was saved (despite post-operative irradiation in six instances). We now believe that when such drastic treatment is deemed necessary operation should seldom be advised ". We would take an intermediate view. We do not think that a " more aggressive surgical approach " is capable of making an appreciable difference to the results in any tumour of very high malignancy, which unfortunately most carcinomas of the parotid are. On the other hand, a tumour which at the time of operation is thought to be a carcinoma, sometimes even after preliminary frozen section biopsy, may prove on full examination to be a tumour of intermediate malignancy such as a mucoepidermoid tumour. Failure to perform the necessary radical removal in such a case robs the patient of an excellent chance of cure. And even with frank carcinomas, we have as already noted four cases surviving without evidence of further growth for from 4 to 6½ years after radical parotidectomy. Our experience with more extensive procedures and with block dissection of the neck would lend some support to Mustard and Anderson's argument, but in spite of this we would find it difficult in similar future cases to avoid attempting to rid the patient of his disease if it seemed reasonably possible to do so. And after all, at present such procedures offer the patient the only hope, however small.

The prognosis in the groups which we have classified as mucoepidermoid tumours, cylindromatous tumours and acinic cell tumours is much better. Since there is a clear-cut division in clinical malignancy between, on the one hand, spheroidal, spindle, adeno and squamous cell carcinoma, and on the other, acinic cell tumours, mucoepidermoid tumours, and cylindromatous tumours, there would seem to be an advantage in a corresponding clear cut division in terminology. We have avoided the term mucoepidermoid carcinoma, and preferred the term cylindromatous tumour to the alternative of adenoid cystic carcinoma. We would suggest that this terminology should be extended and the third category of this intermediate malignant group termed "acinic cell tumour". The term carcinoma would thus be reserved for a group of tumours of widely varying cellular structure, but having the general behavioural characteristic of a high degree of malignancy. The results of treatment of these intermediate tumours have almost certainly been rendered worse by the very limited removal of parotid tissue which was the standard policy not many years ago. The general adoption of the policy of wide and early removal of parotid tumours should show dividends in the form of improved results even more so for these tumours of lesser malignancy than in mixed tumours.

A feature of the *acinic cell tumours* is the variable incidence reported by different authors (Table XI).

All four patients in the present series were women, and where sex incidence has been quoted, there has been a marked preponderance of women (Godwin *et al.*, 1954 ; Grage *et al.*, 1961). These tumours are of comparatively low malignancy, both Eneroth (1964) and Abrams *et al.* (1965) in their large series finding five year survivals of 89%.

*Mucoepidermoid tumours* form 15% of the present series, a figure in close agreement with those given by Nanson (1960), Kirklin *et al.* (1951) and Sharp and Helsper (1960). Several other series however report figures in the region of 30%, the variation being due probably to varying criteria for histological diagnosis.

Mucoepidermoid tumours have been recognised as a separate group of salivary neoplasm for twenty years (Stewart *et al.*, 1945), and were originally subdivided into relatively benign and malignant forms. Woolner *et al.* (1954) recognised two such groups, calling them mucoepidermoid tumours and mucoepidermoid carcinomas respectively. Other workers (Foote and Frazell, 1954) and (Sharp and Helsper, 1960) found the need for a third intermediate group, whilst others recognised that a continuous spectrum of malignancy exists. Like Gray *et al.* (1960) we have preferred to keep all these tumours under one main heading, while giving an indication of histological grade. The majority of mucoepidermoid neoplasms fall into a low grade category and, whilst having a recurrence rate higher than mixed tumours, behave for the most part in a benign fashion. However, just such tumours have occasionally given rise to metastases and even caused death, thus showing their malignant potential. The behaviour of the high grade malignant tumour is somewhat more variable, though figures are usually small. Woolner *et al.* (1954) who grouped highly anaplastic tumours separately as undifferentiated carcinoma noted that these mucoepidermoid tumours behaved as fairly high grade carcinomas and the three cases of Grage and Lober (1962) died within two years despite vigorous therapy.

*Cylindromatous tumours* were first described many years ago but only in the last twenty years has there been general recognition that they made up one of the main groups of salivary gland malignancy (Quattlebaum *et al.*, 1946). Cylindromas are relatively commoner in the submaxillary and minor salivary glands than in the parotid, in which they make up approximately one sixth of the malignant tumours. The alternative name of adenoid cystic carcinoma has received increasing support since its adoption by Foote and Frazell in 1954. These tumours, from the reported series and our experience, appear to occur predominantly in women (McCabe and Boles, 1962). The experience of most authors bears out the potential of cylindromas for slow but relentless infiltrating growth ; they are seldom rapidly fatal, and there is usually a long history of multiple recurrences before generalised metastases appear.

In any large series of mixed parotid tumours there are bound to be a few which, histologically, give rise to anxiety and a suspicion of malignancy. In our series there were only 6 *tumours with suspicious local areas* out of 463 mixed tumours. Very rarely cases are recorded in which a typical mixed tumour, usually after a long history with local recurrences, metastasises to a distant site, the metastasis looking histologically just like the parotid primary (Kirklin, 1951 ; Thackray, 1957). This is the only type of tumour to which we consider the name malignant mixed tumour can reasonably be applied.

The clinical follow-up of our cases has shown no evidence of malignancy but the suspicion remains that the atypical cell changes seen may herald the evolution of more sinister pathology. Just such a change appears to have taken place in Case 2 of Thomas and Cappola (1965).

There are fewer figures available in the literature for the incidence of *malignant lymphomas* than for the primary epithelial malignancies of the parotid. Furthermore the group of benign lympho-epithelial lesions described by Godwin (1952) and other possibly related lymphocytic infiltrations of the parotid have undoubtedly caused difficulty in the histological diagnosis of malignant lymphoma in the past and indeed still do occasionally. This has been emphasised recently by Cruickshank (1965) in a review of eight cases of benign lymphoepithelial lesion of the parotid. He states that in several cases there was anxiety as to whether the lesion might be a lymphosarcoma, particularly those showing distortion and without lymphoid follicles. This uncertainty is heightened when only small biopsy fragments are available for microscopy.

Our group of *secondary tumours* in the parotid were all considered at the time of operation to be primary parotid tumours, but with the exception of the malignant melanoma, all proved eventually to be metastases from distant deep primary tumours. Malignant melanomas feature in most series of parotid tumours and are probably intraparotid glandular secondaries from facial lesions. The series of Grage and Lober contains a somewhat higher incidence of secondary tumours, 10%, but they had a number of cases with primary sites on the face and head which would probably have given rise to a clinical suspicion of the secondary nature of the parotid neoplasm. Our cases, again excepting the fairly recent malignant melanoma, all died of carcinomatosis, whereas the report of Grage and Lober emphasises that with a local primary site metastatic neoplasm " does not necessarily indicate a hopeless situation ".

In conclusion, although there is now much more known about the various types of parotid tumour than in Sir John Bland-Sutton's time, what he wrote in " Tumours, Innocent and Malignant " is still to some extent true : " These tumours are a pathological puzzle and a source of much unsatisfactory speculation ". Incidentally, on the page facing that from which the above quotation is taken is a picture of a woman with a parotid tumour which had grown slowly for 17 years and then " when the woman was 57 it grew quickly, infected the lymph nodes, and the patient died ". One of the main objects of this paper is to emphasise that a fatality such as this can be prevented by complete removal of the original tumour.

SUMMARY

1. The histological material from 95 cases of malignant disease of the parotid treated at the Middlesex Hospital from January 1930 to August 1964 has been re-examined, and an attempt made to correlate the clinical course with the histological appearances.

2. During the same period, 548 other parotid tumours were removed in the hospital, giving a ratio of malignant to other tumours of 1 to 5·8.

3. Most of the 47 patients with tumours classified as carcinoma have died of the disease, and differences in the clinical course between the different cell types of carcinoma are minor.

4. Most of the 32 patients with tumours classified as mucoepidermoid, cylindromatous, and acinic cell tumours have remained well and free of disease.

5. In 18 cases there was histological evidence of the development of the carcinoma in relation to pre-existing primary or recurrent mixed tumours, and in 2 cases to cylindromatous tumours. In addition, there were 5 cases with a history of inert tumour for 10 years or more before the development of the carcinoma. Thus in 25 out of the 47 cases of carcinoma there was either histological or clinical evidence of the origin of the tumour in relation to a tumour of lesser malignancy.

6. All 6 patients with tumours classified as "mixed tumour with suspicious local area" remained well and free of disease.

7. There were 6 cases of secondary tumours of the parotid, one a lymph-borne melanoma, and the other 5 blood-borne tumours. In 2 of the latter, the tumours of the parotid were regarded as primary both clinically and pathologically, and the correct diagnosis was only made in light of the post-mortem findings.

8 Correct surgical treatment of the primary tumour should cure most cases of mucoepidermoid, cylindromatous, and acinic cell tumours.

9. The only immediate hope for substantial improvement in the results of treatment of carcinoma of the parotid is by a reduction of its incidence by adequate surgical treatment of tumours of lesser degree of malignancy.

The authors wish to gratefully acknowledge receipt of a grant from the British Empire Cancer Campaign for Research for the expenses of this work.

### REFERENCES

ABRAMS, A. M., CORNYN, J., SCHOFIELD, H. H. AND HANSEN, L. S.—(1965) *Cancer, N.Y.*, **18**, 1145.

BAUER, W. H. AND BAUER, J. D.—(1953) *Archs Path.*, **55**, 328.

BEAHRS, O. H., WOOLNER, L. B., CARVETH, S. W. AND DEVINE, K. D.—(1960) *A.M.A. Archs Surg.*, **80**, 890.

*Idem*, WOOLNER, L. B., KIRKLIN, J. W. AND DEVINE, K. D.—(1957) *Ibid.*, **75**, 605.

BISHOP, E. L.—(1960) *J. med. Ass. Ga*, **49**, 573.

BLAND-SUTTON, J.—(1922) 'Tumours Innocent and Malignant'. 7th Edition. London (Cassell), p. 414.

BRUZELIUS, S., CEDERQUIST, E., LINELL, F. AND BERGMAN, F.—(1957) *Acta chir. scand.*, **114**, 1.

BUXTON, R. W., MAXWELL, J. H. AND FRENCH, A. J.—(1953) *Surgery Gynec. Obstet.*, **97**, 401.

BYARS, L. T., ACKERMAN, L. V. AND PEACOCK, E.—(1957) *Ann. Surg.*, **146**, 40.

CRUICKSHANK, A. H.—(1965) *J. clin Path.*, **18**, 391.

DARGENT, M.—(1952) *Acta Un. int. Cancr.*, **8**, 359.

ENEROTH, C. M.—(1964) *Acta oto-lar.*, Suppl. R.1.

FOOTE, F. N. AND FRAZELL, E. L.—(1954) 'Tumours of the Major Salivary Glands'. Atlas of Tumour Pathology, U.S. Armed Forces Inst. of Path., Sect. IV., Fasc. 11.

GODWIN, J. T.—(1952) *Cancer N. Y.* **5**, 1089
*Idem*, FOOTE, F. W. AND FRAZELL, E. L.—(1954) *Am. J. Path.*, **30**, 465.
GRAGE, T. B. AND LOBER, P. H.—(1962) *Surgery, St Louis*, **52**, 284.
*Iidem* AND ARHELGER, S. W.—(1961) *Am. J. Surg.*, **102**, 765.
GRAY, J. M., HENDRIX, R. C. AND FRENCH, A. J.—(1963) *Cancer, N.Y.*, **16**, 183.
HAMPERL, H. AND HELLWEG, G.—(1957) *Ibid.*, **10**, 1187.
HANNA, D. C. AND GAISFORD, J. C.—(1962) *Am. J. Surg.*, **104**, 737.
JOHNSON, J. K. AND CHILDERS, J. H.—(1954) *Tex. Rep. Biol. Med.*, **12**, 979.
KIRKLIN, J. W., MCDONALD, J. R., HARRINGTON, S. W. AND NEW, A. B.—(1951) *Surgery Gynec. Obstet.*, **92**, 721.
MCCABE, B. F. AND BOLES, R.—(1962) *Ann. Otal. Rhinol. Lar.*, **71**, 448.
MUSTARD, R. A. AND ANDERSON, W.—(1964) *Ann. Surg.*, **159**, 291.
NANSON, E. M.—(1960) *Ann. R. Coll. Surg.*, **26**, 157.
PATEY, D. H. AND THACKRAY, A. C.—(1958) *Br. J. Surg.*, **45**, 477.
QUATTLEBAUM, F. W., DOCKERTY, M. B. AND MAYO, C. W.—(1946) *Surgery Gynec. Obstet.*, **82**, 342.
RANGER, D., THACKRAY, A. C. AND LUCAS, R. B.—(1956) *Br. J. Cancer*, **10**, 1.
RAWSON, A. J., HOWARD, J. M., ROYSTER, H. P. AND HORN, R. C.—(1950) *Cancer, N.Y.*, **3**, 445.
SHARP, G. S. AND HELSPER, J. T.—(1960) *Calif. Med.*, **93**, 187.
SLAUGHTER, D. P., SOUTHWICK, H. W. AND WALTER, L.—(1953) *Surgery Gynec. Obstet.*, **96**, 535.
STEWART, F. W., FOOTE, F. W. AND BECKER, W. F.—(1945) *Ann. Surg.*, **122**, 820.
THACKRAY, A. C.—(1957) " Pathology of Malignant Tumours Salivary Glands " *in* ' Cancer ' (Butterworth & Co., Ltd., London), Vol. 2.
*Idem* AND LUCAS, R. B.—(1960) *Br. J. Cancer*, **14**, 612.
THOMAS, W. H. AND COPPOLA, E. D.—(1965) *Am. J. Surg.*, **109**, 724.
WOOLNER, L. B., PETTET, J. R. AND KIRKLIN, J. W.—(1954) *Am. J. clin. Path.*, **24**, 1350.